# Toward Convenient and Accurate IMU-Based Gait Analysis

**DOI:** 10.3390/s25041267

**Published:** 2025-02-19

**Authors:** Mohamed Boutaayamou, Doriane Pelzer, Cédric Schwartz, Sophie Gillain, Gaëtan Garraux, Jean-Louis Croisier, Jacques G. Verly, Olivier Brüls

**Affiliations:** 1 Laboratory of Movement Analysis, University of Liège, B-4000 Liège, Belgium; cedric.schwartz@uliege.be (C.S.); jlcroisier@uliege.be (J.-L.C.); o.bruls@uliege.be (O.B.); 2 Department of Electrical Engineering and Computer Science, University of Liège, B-4000 Liège, Belgium; jacques.verly@uliege.be (J.G.V.); 3 Physical Medicine and Sport Traumatology Department, University Hospital of Liège, B-4000 Liège, Belgium; dpelzer@chuliege.be (D.P.); 4 Geriatrics Department, University Hospital of Liège, B-4000 Liège, Belgium; sgillain@uliege.be (S.G.); 5 Neurology Department, University Hospital of Liège, B-4000 Liège, Belgium; ggarraux@uliege.be (G.G.)

**Keywords:** gait analysis, accelerometers, gyroscopes, inertial sensors, spatiotemporal parameters, signal-processing algorithms, reference data

## Abstract

While inertial measurement unit (IMU)-based systems have shown their potential in quantifying medically significant gait parameters, it remains to be determined whether they can provide accurate and reliable parameters both across various walking conditions and in healthcare settings. Using an IMU-based system we previously developed, with one IMU module on each subject’s heel, we quantify the gait parameters of 55 men and 46 women, all healthy and aged 40–65, in normal, dual-task, and fast walking conditions. We evaluate their intra-session reliability, and we establish a new reference database of such parameters showing good to excellent reliability. ICC(2,1) assesses relative reliability, while SEM% and MDC% assess absolute reliability. The reliability is excellent for all spatiotemporal gait parameters and the stride length (SL) symmetry ratio (ICC ≥ 0.90, SEM% ≤ 4.5%, MDC% ≤ 12.4%) across all conditions. It is good to excellent for the fast walking performance (FWP) indices of stride (Sr), stance (Sa), double-support (DS), and step (St) times; gait speed (GS); and the GS normalized to leg length (GS_n1_) and body height (GS_n2_) (ICC ≥ 0.91, |SEM%| ≤ 10.0%, |MDC%| ≤ 27.6%). Men have a higher swing time (Sw) and SL across all conditions. The following parameters are gender-independent: (1) Sa, DS, GS_n1_, GS_n2_; (2) the symmetry ratios of SL and GS, as well as Sa% and Sw% (representing Sa and Sw as percentages of Sr); and (3) the FWPs of Sr, Sa, Sw, DS, St, cadence, Sa% and Sw%. Our results provide reference values with new insights into gender FWP comparisons rarely reported in the literature. The advantages and reliability of our IMU-based system make it suitable in medical applications such as prosthetic evaluation, fall risk assessment, and rehabilitation.

## 1. Introduction

The gait of a person is the manner in which this person walks or, more generally, moves on their feet. Gait analysis refers to the analysis of this gait, and the methods for performing this analysis can be broadly classified into visual methods and instrumental methods, with the latter ones providing numerical parameters (often spatiotemporal in nature), and thus providing a better description of the gait [1]. These numerical (and thus quantitative) parameters may guide healthcare professionals in making detailed diagnoses and optimal treatment decisions for conditions that impact the ability of a patient to walk (e.g., [2,3,4,5,6]).

Instrumental methods rely on advanced systems consisting, in a broad way, of hardware, control software, and signal-processing algorithms. Very often, these systems are cumbersome and expensive, and thus available only in well-equipped environments, thereby necessarily preventing their widespread use in the typical clinical contexts [7]. Advancements in microelectronics have led to the development of small devices containing inertial sensors (namely accelerometers and gyroscopes) providing (raw) inertial measurement signals, with each such device being commonly called an “inertial measurement unit (IMU)”. These small and lightweight IMUs can easily be integrated in wearables, which can be used as the basis of the gait analysis systems used in healthcare settings (e.g., [8]), furthermore saving space, labor, and time. These IMU-based wearable systems can also record gait data over extended and continuous time periods, overcoming the limitations of the measurement volume typically encountered in some systems like instrumented mats and motion capture setups.

While previous research using IMU-based systems for gait analysis has shown promising results in quantifying key gait features/parameters (e.g., [9,10,11]), significant work remains to be performed to support the use of these systems in clinical settings [12]. In particular, there is a lack of studies that thoroughly evaluate the reliability of the gait parameter values provided by these systems across different walking speeds, while accounting for gender, leg length, and body height characteristics in a large sample of healthy adults [10,13]. The accuracy and reliability of these parameters has, however, to be examined before they can be used in clinical gait studies. Reliability refers to the consistency or reproducibility of accurate measurements when repeated for the same participant. Intra-session reliability refers to the degree of consistency in measurement outcomes across repeated trials performed within the same session.

Additionally, fewer studies provide reference values for gait parameters quantified using IMU-based systems. It is, however, crucial to establish such reference values and to verify their consistency with existing normative gait data; this would then support the use of IMU-based systems in clinical studies. When applying these systems to assess an abnormal gait, the extracted parameters can help quantify deviations from normal gait patterns. Moreover, establishing reference parameters that account for participant characteristics could improve the accuracy of medical diagnoses and enhance the evaluation of responses to gait rehabilitation.

The aim of the present paper is, therefore, twofold: (1) to thoroughly evaluate the intra-session reliability of various gait parameters and compare the results with those from previous studies, and (2) to establish reference values for these parameters, specific to gender, leg length, and body height, during over-ground walking at three different speed conditions: preferred, dual-task, and fast walking. These parameters include 15 spatiotemporal gait parameters; the four symmetry indices of ten spatiotemporal gait parameters; the dual-task cost; and the fast walking performance indices of 15 spatiotemporal gait parameters. The three speed conditions are considered to provide a comprehensive assessment of an individual’s walking performance: (1) preferred walking establishes a baseline of the normal walking pattern, (2) dual-task walking evaluates cognitive–motor integration under divided attention, and (3) fast walking determines the maximal functional capacity. Furthermore, the work described here considers individuals aged 40 to 65 years to cover a representative sample of middle-aged adults and to quantify IMU-based reference gait parameters while minimizing the confounding effects of age-related changes. For instance, gait speed has been shown to begin declining at age 65 [14]. The chosen age range aligns with established reference gait parameters for this group and enables meaningful comparisons with other similar studies in the existing literature [15,16,17,18,19,20].

The major contributions of this work include quantifying and establishing the reliability level of some key reference gait parameters not readily available in the literature. To our knowledge, this is the first study to obtain these parameters by analyzing gait signals measured from IMUs attached to the heels of regular shoes, using dedicated and validated signal-processing algorithms.

Our work is organized as follows: Section 2 successively explains how we determined the sample size for the participants we recruited in this study, describes the IMU-based hardware system and its use in quantifying gait parameters, details our experimental procedure, defines the considered gait parameters, and explains how we assess their reproducibility. Section 3 presents the results of the relative and absolute reliability of these parameters and provides a reference database of parameters demonstrating good to excellent reliability. Section 4 reviews the obtained results, compares them to previous studies, provides insights into advancements in IMU-based gait analysis, discusses study limitations, and suggests directions for future research. Section 5 summarizes our findings and gives our conclusions.

## 2. Materials and Methods

### 2.1. Participants

We used the G*Power software (version 3.1.9.7; universities of Kiel, Düsseldorf, and Mannheim, Germany) [21] to calculate the sample size required for groups of healthy women and men aged 40–50 years. We conducted a pilot study that provided the following mean and SD values for walking speed [m/s]: 1.469 ± 0.170 and 1.559 ± 0.118 at PW, 1.409 ± 0.184 and 1.629 ± 0.278 at DTW, and 1.927 ± 0.252 and 2.100 ± 0.261 at FW; this was in 10 healthy men and 10 healthy women aged 40–65 years, respectively. Using a 2-tailed test with an alpha of 0.05, the calculated sample sizes per group needed to achieve a power of at least 0.8 were 43 for PW, 20 for DTW, and 36 for FW. We therefore aimed to recruit at least 43 participants per group.

This effort resulted in a total of 46 healthy women and 55 healthy men who agreed to participate in the study (Table 1). They were able to walk without any musculoskeletal pain and had no history of hip or knee prostheses or neurologic disorders. The local ethics committee of the University Hospital of Liège, Belgium, approved the study protocol.

### 2.2. IMU-Based Hardware System

To record the raw gait signals, we used a stand-alone hardware system that is based on commercially available IMU modules and that we developed, designed, and implemented at the University of Liège (ULiège), Belgium [22], including the hardware, control software, and signal-processing algorithms. The hardware consists of (1) a central unit with memory, a microcontroller, and a battery, (2) four small IMU modules (2 cm × 0.7 cm × 0.5 cm), and (3) four wires connecting the IMUs to the central unit.

**Figure 1:**
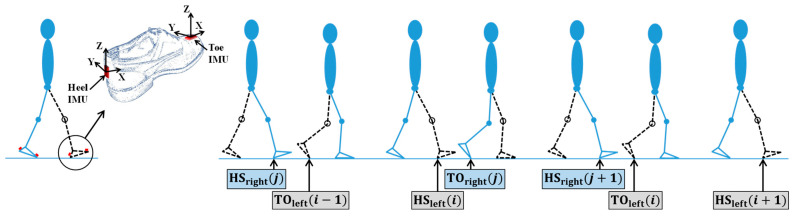
(*Left*) We use the stand-alone hardware system to record gait signals from four IMUs tightly attached to the participants’ regular shoes: two at the level of the left and right heels, and two at the level of the left and right toes. We only consider raw gait signals from the (two) heel IMUs to extract reference values for spatiotemporal gait parameters. (*Right*) Schematic illustration of consecutive and overlapping left gait cycles *i* and right gait cycles *j* from which the signal-processing algorithms accurately and precisely extract the (left and right) heel strike (HS) and toe-off (TO) timings involved in the calculation of the spatiotemporal gait parameters.

The central unit is positioned at the waist. For each test, we attach in a rigorous and systematic way two IMU modules to each shoe of each participant, one at the toe and one at the heel (Figure 1), for a total of four modules for each participant.

The hardware system operates in a wired configuration, without wireless-connected IMUs. We took specific measures to mitigate any unwanted bias that the wires might introduce during the gait experiments. For instance, we used lightweight and flexible wires that are sufficiently long to allow participants to move freely. Additionally, these wires were securely fixed behind the legs to prevent mechanical interference with the participants’ movements. After each gait session, we systematically asked participants whether the wires caused any discomfort or impeded their movement, and all participants reported that the wires neither caused discomfort nor interfered with their movements. Moreover, the IMU modules were tightly attached to prevent any misalignment or relative movement between the IMUs and the shoes. We performed systematic checks during the processing of the recorded gait data and found no artifacts in the raw data.

An oversampling was applied to the IMUs, with the microcontroller functioning as a master that sequentially read the values from each IMU. Given the wired setup, challenges related to clock synchronization in wireless systems, as discussed in [23], were not applicable to our system.

The system measures 3-axis accelerations (up to ±16 g) and 3-axis angular velocities (up to ±2000°/s). To extract reference values for the gait parameters, we only used the raw signals from the two heel IMUs in this work. Specifically, the analysis of the signals from the toe IMUs is beyond the scope of this study and will be considered in future work (see Section 4.4).

### 2.3. Experimental Procedure

Participants wore their own regular shoes (excluding sandals and high heels). Before data recording, they performed one warm-up trial at their self-selected speed. Each participant completed two consecutive gait trials, denoted here by trial1 and trial2, along a 30 m distance in a wide, clear, straight hallway at (1) preferred walking (PW), (2) dual-task walking (DTW), and (3) fast walking (FW) speeds.

We added 3 m to the nominal 30 m to allow for the exclusion of the first and last two strides during the processing of the gait signals, which minimizes the effects of the periods of gait initiation/termination, acceleration, and deceleration [12]. We focus here on the analysis of the intra-session reliability of the parameter reference values extracted during the steady-state walking periods in trial1 and trial2.

For FW, we asked the participants to walk as fast and safely as possible without running. To assess the effect of a concurrent task on gait, DTW included a cognitive task, namely “serial sevens subtractions” [24], where the subject must announce in an audible voice the results of subtracting seven from a starting number while walking. Participants were instructed to prioritize neither the gait nor the cognitive task. We conducted all the gait tests at the Laboratory of Movement Analysis of ULiège.

### 2.4. Quantification of Gait Parameters

From the two raw, time-synchronized signals from the two heel IMUs, we extracted the spatiotemporal gait parameters by using the method that we describe in [25], where we successively (1) parsed heel acceleration data into flat and non-flat phases, and (2) applied an appropriate signal-processing algorithm to the acceleration sub-signals delimited by the non-flat phases to identify heel strike (HS) and toe-off (TO) timings [25]. This algorithm uses distinctive and remarkable features in these sub-signals to extract HSs and TOs with good accuracy and precision [26].

Accuracy and precision correspond to the averages of the mean and standard deviation (SD), respectively, of the (signed) differences between the IMU-derived method and reference method timings (e.g., timings from methods based on kinematic and force plates). For instance, the accuracy ± precision values for HS and TO are, respectively, 1 ms ± 12 ms and 0 ms ± 7 ms for older adults during the comfortable walking condition [27]. The individual values of the gait parameters are computed using the HS and TO in consecutive and overlapping left gait cycles *i* and right gait cycles *j*, as summarized in Table 2 and illustrated in Figure 1.

In [27], we show that the accuracy ± precision values are the following for Sr: 0 ms ± 15 ms, Sa: 0 ms ± 14 ms, Sw: 0 ms ± 14 ms, and DS: 0 ms ± 14 ms. Additionally, we use the method in [22] to quantify the individual stride lengths and gait speeds. This method robustly detects zero-velocity update regions in the gait signals and applies adequate initial conditions to minimize integration drifts during successive strapdown integrations at the level of individual strides. This method yields an accuracy and precision of −0.7 ± 4.4 cm for SL, and −6.7 ± 6.7 cm/s for GS, during preferred walking.

We assess the differences between gait parameters from trial1 and trial2, for each of the three walking conditions, by first using the Shapiro−Wilk parametric test to check whether the corresponding distribution is normal (i.e., Gaussian) or not, and by then using the Student t-test for normal distributions and the nonparametric Wilcoxon rank sum test otherwise. The mean and SD values of the gait parameters from the left and right sides are calculated for intra- and inter-participants. Table S1 shows there are no significant differences between the left and right values. We then provide all the gait parameter values as the mean and SD of the combined left and right gait parameters.

SL and GS are divided by the leg length and body height, yielding, respectively, the normalized parameters SL_n1_ [dimensionless], GS_n1_ [s^−1^], SL_n2_ [dimensionless], and GS_n2_ [s^−1^] [28]. The leg length is calculated as the average of the left/right leg lengths as no significant difference is found when comparing the left leg lengths to the right ones.

We also examine the intra-session reliability of dimensionless symmetry indices, namely the symmetry index (SI1), symmetry ratio (SI2), symmetry angle (SI3), and an alternative version of the symmetry ratio (SI4). Since we have access to gait parameters extracted on a stride-by-stride basis, we calculate these quantities as the mean of individual symmetry indices $I_{k}$ using four commonly reported formula (e.g., [29]), as follows:

(1) $$SI1=\frac{1}{n}\sum_{k=1}^{n}I_{k}\;,\;\;with\;I_{k}=\frac{1}{m}\sum_{i=1}^{m}100\frac{\left|X_{D}\left(i\right)-X_{nD}\left(i\right)\right|}{0.5\;\left(X_{D}\left(i\right)+X_{nD}\left(i\right)\right)}\;\;,$$

(2) $$SI2=\frac{1}{n}\sum_{k=1}^{n}I_{k}\;,\;\;with\;I_{k}=\frac{1}{m}\sum_{i=1}^{m}\frac{\min\left(X_{D}(i),X_{nD}(i)\right)}{\max\left(X_{D}(i),X_{nD}(i)\right)}\;\;,$$

(3) $$SI3=\frac{1}{n}\sum_{k=1}^{n}I_{k}\;,\;\;with\;I_{k}=\frac{1}{m}\sum_{i=1}^{m}100\frac{45{}^{\circ}-\mathrm{atan}\left(\frac{X_{nD}\;(i)}{X_{D}\;(i)}\right)}{90{}^{\circ}}\;\;,$$

(4) $$SI4=\frac{1}{n}\sum_{k=1}^{n}I_{k}\;,\;\;with\;I_{k}=\frac{1}{m}\sum_{i=1}^{m}\frac{X_{nD}(i)}{X_{D}(i)}\;\;,$$

where $X_{nD}\;\left(i\right)$ and $X_{D}($*i*) are individual values of a gait parameter from the non-dominant (nD) and dominant (D) sides, respectively, *n* is the total number of participants, and *m* is the smallest value of the total numbers of left parameters and right parameters for a given participant. The ability of a participant to handle a second task while walking is characterized using the dual-task cost (DTC) with

(5) $$DTC=X_{DTW}-X_{PW}\;,$$

(6) $$DTC\%=100(X_{DTW}-X_{PW})/X_{PW\;}\;,$$

where $X_{DTW}$ and $X_{DTW}$ are the average values of a gait parameter from the DTW and PW tests, respectively. Analogously, we define the fast walking performance index (FWP) as

(7) $$FWP=X_{FW}-X_{PW}\;,$$

(8) $$FWP\%=100{(X}_{FW}-X_{PW})/X_{PW\;}\;,$$

where $X_{FW}$ is the average value of a gait parameter from the FW tests.

### 2.5. Reproducibility Analysis of Gait Parameters

This paper examines the relative and absolute intra-session reliability of gait parameters from trial1 and trial2 across the three walking conditions.

We use the intraclass correlation coefficient ICC(2,1) and its 95% confidence interval (95% CI) to estimate the relative intra-session reliability [30]. Moreover, we adopt the following interpretation of ICCs to evaluate the level of relative intra-session reliability [31]:

$$\mathrm{ICC interpretation}=\left\{\begin{array}{l} \mathrm{Poor},\;\;\;\;\;\;\;\;\;\;\;\;\;\;\;\;\;\;\;\;\;\;\;\;\;\;\mathrm{if}\;\mathrm{ICC}<\;0.50\\ \mathrm{Moderate},\;\mathrm{if}\;0.50\;\leq \;\mathrm{ICC}\;<\;0.75\\ \mathrm{Good},\;\;\;\;\;\;\;\;\;\mathrm{if}\;0.75\;\leq \;\mathrm{ICC}\;<\;0.90\\ \mathrm{Excellent},\;\;\;\;\;\;\;\;\;\;\;\;\;\;\;\;\;\mathrm{if}\;\mathrm{ICC}\;\geq \;0.90 \end{array}\right.$$

In addition, we use the standard error of measurement (SEM) and minimal detectable change (MDC) to estimate the absolute intra-session reliability. The SEM measures the absolute reliability by estimating the variation in measurement errors [32]. It is calculated as $SEM=SD\sqrt{1-r}$, where SD is the standard deviation of the gait parameter across participants, and r is the reliability coefficient (i.e., ICC(2,1) here). Smaller SEM values indicate a higher absolute reliability. The SEM estimates how repeated measurements of a participant’s gait parameter are distributed around the true value. The MDC is the smallest measurement change value above which a real change has occurred (e.g., [33]); it is calculated as $MDC=1.96\sqrt{2}\;SEM$. The SEM is multiplied by 1.96 to determine the 95% CI, and by $\sqrt{2}$ for repeated measurement error adjustment [34]. Both SEM and MDC are also expressed as percentages of the gait parameter mean: SEM% and MDC%.

The literature lacks clear criteria for evaluating the absolute intra-session reliability. One should note, however, that SEM% and MDC% are related to the coefficient of variation (CV=100 SD/mean) using the following formula: $SEM\%=CV\sqrt{1-r}$ and $MDC\%=1.96\sqrt{2}CV\sqrt{1-r}$. Since $0\leq \sqrt{1-r}\leq 1$ (as |r|≤1), we have |SEM%|≤|CV| and $\left|MDC\%|\leq 1.96\sqrt{2}\;|CV\right|$. In addition, |CV|>20% can be considered as poor, 10%<|CV|≤20%: moderate, 5%<|CV|≤10%: good, and |CV|≤5%: excellent. Assuming these CV’s cut-offs, we propose the following criteria to evaluate the level of absolute intra-session reliability:

$$\mathrm{SEM}\%,\;\mathrm{MDC}\%\;\mathrm{interpretation}=\left\{\begin{array}{cc} \mathrm{Poor}, & \;\;\;\;\;\;\;\;\;\;\;\;\;\;\;\;\;\;\;\;\;\;\;\;\;\;\;\;\;\;\;\;\;\;\;\;\;\mathrm{if}\;|\mathrm{SEM}\%|>20\%\;\mathrm{or}\;|\mathrm{MDC}\%|>60\%\\ \mathrm{Moderate}, & \;\mathrm{if}\;10\%<|\mathrm{SEM}\%|\leq 20\%\;\mathrm{or}\;30\%<|\mathrm{MDC}\%|\leq 60\%\\ \mathrm{Good}, & \;\;\;\;\;\;\;\;\;\;\mathrm{if}\;5\%<|\mathrm{SEM}\%|\leq 10\%\;\mathrm{or}\;15\%<|\mathrm{MDC}\%|\leq 30\%\\ \mathrm{Excellent}, & \;\;\;\;\;\;\;\;\;\;\;\;\;\;\;\;\;\;\;\;\;\;\;\;\;\;\;\;\;\;\;\mathrm{if}\;|\mathrm{SEM}\%|\leq 5\%\;\mathrm{or}\;|\mathrm{MDC}\%|\leq 15\% \end{array}\right.$$

The Bland–Altman plots provide the 95% limits of agreement s(LOA) for the intra-session gait parameters. The data are analyzed using Matlab (R2018b, MathWorks, Natick, MA, USA) and the significance level is set at a *p*-value of 0.05.

## 3. Results

We quantified the gait parameters from a total of 6405, 6804, and 5425 individual strides extracted under the PW, DTW, and FW conditions, respectively, after carefully and visually inspecting all the results from each of the algorithm steps (e.g., the segmentation and the extracted HSs and TOs).

Figures S1−S3 provide the Bland–Altman plots and distributions of individual spatiotemporal gait parameters from each side in trial1 and trial2, for each walking condition. We obtained these distributions by pooling all the individual left/right parameters. The corresponding average values (i.e., 101 values) were obtained and their Bland–Altman plots and distributions are given in Figures S4−S6, all showing values well distributed around zero.

This section places more weight on presenting the results of the relative and absolute reliability of the gait parameters and their reference values.

### 3.1. Reliability of Spatiotemporal Gait Parameters and Symmetry Indices

**Figure 2:**
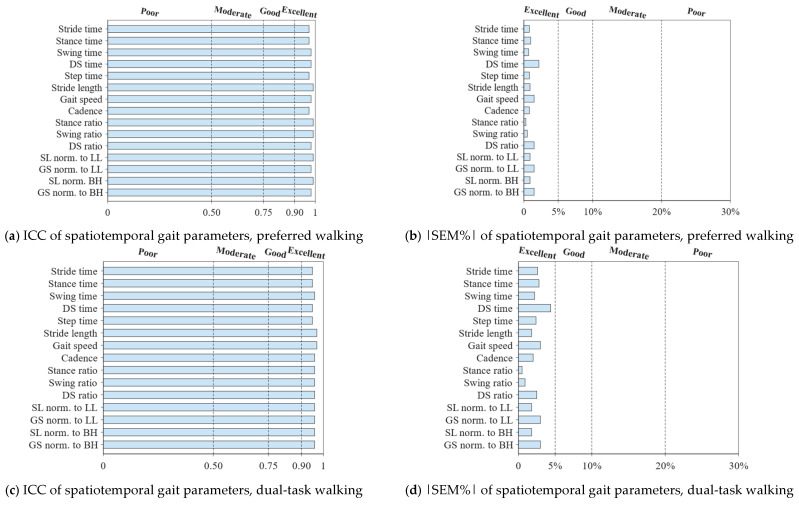

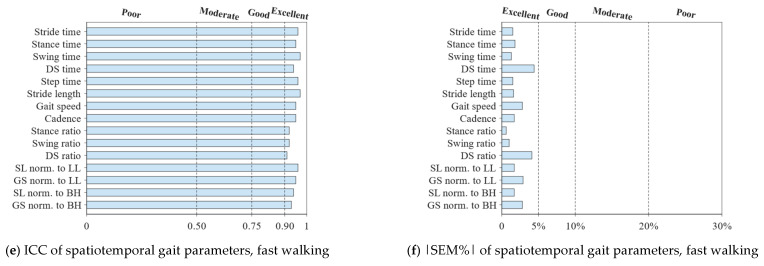
ICC and |SEM%| results for spatiotemporal gait parameters in (**a**,**b**) preferred, (**c**,**d**) dual-task, and (**e**,**f**) fast walking speed conditions. These parameters include *stride time* (Sr) [s], *stance time* (Sa) [s], *swing time* (Sw) [s], *double-support time* (DS) [s], *step time* (St) [s], *stride length* (SL) [m], *gait speed* (GS) [m/s], and *cadence* (Cad) [strides/s]. The *stance*, *swing*, and *DS ratios* are the stance, swing, and DS as percentages [%] of Sr, respectively. SL and GS are divided by leg length (LL) and body height (BH), yielding, respectively, the following normalized parameters: *SL norm. to LL* [dimensionless], *GS norm. to LL* [s^−1^], *SL norm. to BH* [dimensionless], and *GS norm. to BH* [s^−1^]. The dashed lines correspond to the ICC and |SEM%| thresholds, as detailed in Section 2.5.

**Figure 3:**
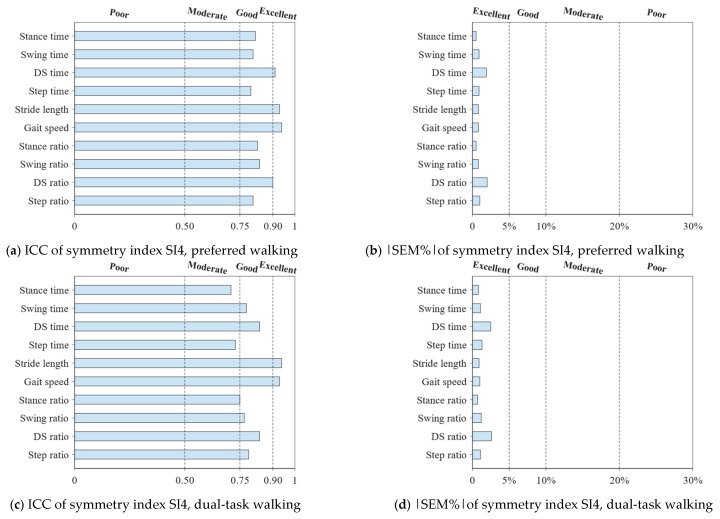

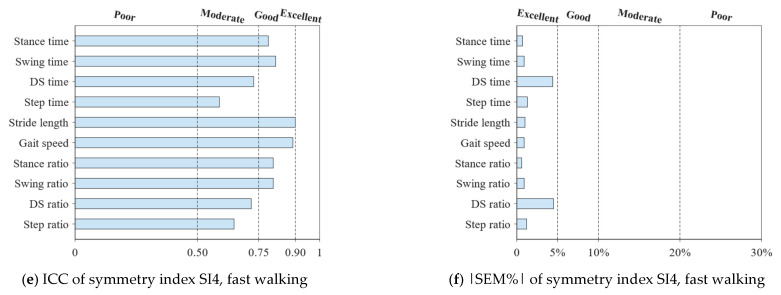
ICC and |SEM%| results for symmetry index SI4 in (**a**,**b**) preferred, (**c**,**d**) dual-task, and (**e**,**f**) fast walking speed conditions. The index values are obtained by applying Formula 4 to *stance time*, *swing time*, *double-support time* (DS), *step time*, their respective percentages (*stance*, *swing*, *DS*, and *step ratios*) of stride time, *stride length*, and *gait speed*. The dashed lines correspond to the ICC and |SEM%| thresholds, as detailed in Section 2.5.

Figure 2 and Figure 3, Table 3 and Table 4, and Tables S2−S4 show the values obtained for the spatiotemporal gait parameters and symmetry indices, and their intra-session reliability at PW, DTW, and FW. No significant differences are found between these values in trial1 and trial2.

The relative reliability is excellent for all spatiotemporal gait parameters (0.92≤ICC<0.99) across all walking conditions (Figure 2 and Table 3). The relative reliability for symmetry indices SI1 and SI2 is moderate across the walking conditions, except for (1) SI1 and SI2 of SL and GS at PW and DTW, showing good reliability, and (2) SI2 of Sa at PW and St and St% at FW, showing poor reliability (Tables S2 and S3). The relative reliability for SI3 (Table S4) and SI4 (Figure 3 and Table 4) is good to excellent at PW, DTW, and FW; this is except for (1) SI3 and SI4 of Sa and St at DTW, and St, DS%, and St% at FW, showing moderate reliability, and except for (2) SI4 of DS at FW, showing moderate reliability.

The absolute reliability is excellent for all spatiotemporal gait parameters across the three walking conditions (Figure 2 and Table 3), with small SEM and MDC values, and SEM% and MDC% not exceeding 4.4% and 12.1%, respectively. The absolute reliability is poor for symmetry indices SI1 (21.5%≤SEM%≤35.1% and 59.5%≤MDC%≤97.2%) and SI3 (94.5%≤|SEM%|≤1145.1% and 262.0%≤|MDC%|≤3174.1%) (Tables S2 and S4). Excellent absolute reliability is, however, found for symmetry indices SI2 (0.3%≤SEM%≤3.1% and 0.9%≤MDC%≤8.6%) (Table S5) and SI4 (0.5%≤SEM%≤4.5% and 1.4%≤MDC%≤12.4%) (Figure 3 and Table 4).

### 3.2. Reliability of Dual-Task Cost and Fast Walking Performance

**Figure 4:**
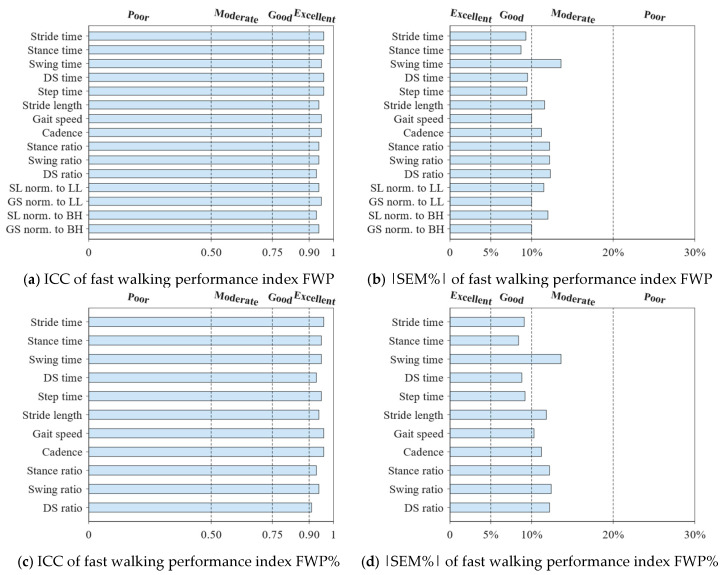
ICC and |SEM%| results of fast walking performance indices, (**a**,**b**) FWP and (**e**,**f**) FWP%. These indices are obtained for *stride time*, *stance time*, *swing time*, *double-support time* (DS), their respective percentages (*stance*, *swing*, and *DS ratios*) of stride time, *step time*, *stride length* (SL), *gait speed* (GS), and *cadence.* SL and GS are divided by leg length (LL) and body height (BH), yielding, respectively, the following normalized parameters: *SL norm. to LL* [dimensionless], *GS norm. to LL* [s^−1^], *SL norm. to BH* [dimensionless], and *GS norm. to BH* [s^−1^]. The dashed lines correspond to the ICC and |SEM%| thresholds, as detailed in Section 2.5.

Figure 4, Table 5 and Table S5 provide the results of the intra-session reliability of the dual-task cost and fast walking performance values. There are no significant differences between these values in trial1 and trial2.

The relative intra-session reliability is excellent for (1) all DTC features (0.90≤ICC≤0.96), except for those of Sa% and Sw% where ICC is good (ICC = 0.89) (Table S5), and (2) all DTC% features (0.91≤ICC≤0.96) except for those of Sa%, Sw%, and DS% where ICC is good (ICC = 0.89) (Table S5). Relative reliability is also excellent for all FWP and FWP% features (0.91≤ICC≤0.96) (Figure 4 and Table 5).

Absolute reliability is poor for DTC and DTC% (45.1%≤|SEM%|≤107.7% and 125.1%≤|MDC%|≤298.6%) (Table S5). The FWP absolute reliability is good for Sr, Sa, DS, St, GS, GS_n1_, and GS_n2_ (8.7%≤|SEM%|≤10.0% and 24.2%≤|MDC%|≤27.6%), and moderate for Sw, SL, Cad, Sa%, Sw%, DS%, SL_n1_, and SL_n2_ (11.2%≤|SEM%|≤13.6% and 31.1%≤|MDC%|≤37.7%) (Figure 4 and Table 5). The FWP% absolute reliability is good for Sr, Sa, DS, and St, (8.4%≤|SEM%|≤9.2% and 23.4%≤|MDC%|≤25.5%), almost good for GS (SEM% = 10.3% and MDC% = 28.4%), and moderate for Sw, SL, Cad, Sa%, Sw%, and DS% (11.2%≤|SEM%|≤13.6% and 31.1%≤|MDC%|≤37.7%) (Figure 4 and Table 5).

### 3.3. Reference Values for Gait Parameters Quantified Using the IMU-Based Method

Table S6 provides the reference values for the spatiotemporal gait parameters and SI4, along with the results of the following parameter comparisons: (1) PW vs. DTW, (2) PW vs. FW, and (3) DTW vs. FW.

The analysis of these results demonstrates the ability of the proposed method to distinguish gait parameters across different walking speed conditions. For instance, the comparison between PW and FW parameters shows that participants significantly increase their stride length from 1.527 m to 1.777 m and significantly reduce their stride duration from 1.041s to 0.886s, leading to a substantial increase in gait speed from 1.471 m/s to 2.016 m/s. Moreover, these changes in parameters include a decrease in DS% from 12.5% to 9.9% and in Sa% from 62.5% to 60.0%, along with an increase in Sw% from 7.5% to 40.0%. Furthermore, the SI4 values for each parameter remain consistent across the three walking speed conditions.

Table 6, Table 7 and Table 8 summarize the results of the gender effect on these reference values and those for the FWP and FWP%. These results indicate that the following parameters are gender-independent (*p* > 0.05) across the three walking conditions: (1) Sa, DS, GS_n1_, and GS_n2_, and (2) SI4 for Sa, Sw, SL, GS, Sa%, and Sw%. Furthermore, there is no effect of gender on FWP and FWP% for Sr, Sa, Sw, DS, St, Cad, Sa%, and Sw%.

## 4. Discussion

The above sections present a validated IMU-based method that (1) assesses the intra-session reliability of various gait parameters in healthy men and women aged 40–65 at three walking conditions, and (2) establishes a new database of reference gait parameters. To the best of our knowledge, this is the first study to obtain these results by analyzing gait signals measured from IMUs attached to the heels of regular shoes, using dedicated and validated signal-processing algorithms.

### 4.1. Reliability of Gait Parameters

The obtained results strongly support previous studies on the intra-session reliability of spatiotemporal gait parameters, which reported similar ICC, SEM (SEM%), and MDC (MDC%) values in healthy participants, mainly at the PW condition. Fewer studies have reported these values at serial sevens DTW and FW conditions.

Table 9 compares some of these values to our results. Similar to [11], SL and SL_n1_ show excellent relative reliability (ICC > 0.90). However, Sa% and Sw% show (1) excellent reliability (ICC = 0.99) here, and (2) moderate reliability (ICC = 0.69) in [11]. Our SEM (SEM%) and MDC (MDC%) values are smaller than those obtained in [11,35] at the PW condition. For instance, the values obtained here are at least five times lower than those found for Sa% and Sw% in [11]. It is important to note that previous studies have employed various modalities, each relying on the manufacturer’s proprietary software, such as Gaitrite [35], the OptoGait portable photoelectric cell system [36], and IMUs placed on dorsal feet [11]. In contrast, we use two IMUs attached to the heels, which results in different gait signals and signal-processing algorithms.

Symmetry indices SI1 and SI3 for the spatiotemporal gait parameters show poor to good relative intra-session reliability, but consistently poor absolute intra-session reliability across all three walking conditions. This may be due to natural gait variability, where slight fluctuations in gait parameters propagate into the calculation of SI1 and SI3, reducing their absolute reliability.

One may use another measure of the dominant/non-dominant symmetry: SI5=100 |ln(nD/D)| [37]. The results of SI1 can, however, be extended to SI5: the series expansion of SI5 around 1 (i.e., when nD/D varies around 1) is 200|nD−D|/(nD+D), which corresponds to SI1.

In addition, the results of SI2 and SI4 for all the gait parameters show excellent absolute intra-session reliability across the three walking conditions. Compared to SI2, SI4 has a better relative intra-session reliability at the three walking conditions. These findings support previous results for SI1 and SI4 at PW, such as those in [11], and provide new insights for SI1, SI2, SI3, SI4, and SI5 at serial sevens DTW and FW conditions, which could aid in the selection of an appropriate symmetry formula to be applied to the adequately chosen gait parameter.

Our work suggests that any future gait study procedure similar to the one presented in Section 2.3 could benefit from using SI4 for (1) Sa, Sw, DS, St, SL, GS, Sa%, Sw%, DS%, and St% at PW, (2) Sw, DS, SL, GS, Sa%, Sw%, DS%, and St% at the serial sevens DTW, and (3) Sa, Sw, SL, GS, Sa%, and Sw% at FW. SI4 has the advantage of being calculated as the average of individual symmetry indices accounting for the dominant/non-dominate sides (Formula (4)), which may reflect the asymmetrical nature of walking, particularly in pathological conditions.

### 4.2. Reliability of Dual-Task Cost and Fast Walking Performance

The DTC and DTC% indices have been used in several dual-task studies to assess the effect of secondary tasks on gait performance, including research on dementia (e.g., [38]), Alzheimer’s disease (e.g., [39]), and fall prediction (e.g., [40]). Fewer studies report, however, their relative and absolute reliability. It is crucial to assess the reliability of these dual-task indices before incorporating them into gait-related medical research.

This study demonstrates that DTC and DTC% exhibit (1) excellent or almost excellent relative reliability (0.89≤ICC≤0.96), and (2) poor absolute reliability across all gait parameters and walking conditions. The SEM% and MDC% values for DTC and DTC% (i.e., 45.1%≤|SEM%|≤107.7%, 124.9%≤|MDC%|≤298.7%) are lower than those reported in [11] (e.g., 68.0%≤|SEM%|≤14994.3% and 188.6%≤|MDC%|≤41562.1%). To investigate the cause of the low relative reliability of these features, we conducted systematic and thorough checks during the processing of the recorded gait data. We found no artifacts in the raw data, nor any errors in the extraction of gait events and parameters. The analysis of individual values of the DTW gait parameter through the Bland–Altman plots reveals that some participants may modify their gait within the same trial and across both trials during the DTW task (see, e.g., these plots for the DTW Sr in Figure S2, page S6). Although we instructed participants to prioritize neither the walking nor the cognitive task—since any prioritization could lead to high variability in their walking—some participants may have used different strategies [41] to handle the cognitive task while walking.

The proposed IMU-based system is, therefore, capable of providing less inflated values of |SEM%| for DTC and DTC% (compared to [11]) and can objectively quantify features that reflect true modifications in gait both within the same trial and across different trials. Based on these results and their comparison with similar studies, the relative reliability of the DTC and DTC% features may primarily stem from natural gait variability.

Previous fast walking studies have used FWP% for various gait parameters, such as investigating the relationship between individuals’ walking speed and lower-limb joint moments using cadence and stride length FWP% [42]. The reliability of the FWP and FWP% has, however, not been assessed so far and should be evaluated before their use in clinical gait studies. To the best of our knowledge, this is the first study to examine this reliability using data obtained from heel-mounted IMUs. In particular, we provided the FWP and FWP% for several spatiotemporal gait parameters showing (1) excellent relative reliability (0.91≤ICC≤0.96), and (2) almost good (10.3%≤|SEM%|≤13.6%) to good (8.4%≤|SEM%|≤9.2%) absolute reliability.

### 4.3. Gender-Based Reference Values for IMU-Derived Gait Parameters

This paper quantifies gender-based reference gait parameters exhibiting almost good to excellent relative and absolute reliability (0.7≤ICC, |SEM%|≤13.6%). These features include the following: (1) 15 parameters at PW, DTW, and FW (0.92≤ICC≤0.99, 0.3%≤SEM%≤4.4%) (Table 6), (2) SI4 of 10 parameters at PW and DTW, and SI4 of 8 parameters at FW (0.71≤ICC≤0.94, 0.5%≤SEM%≤4.5%) (Table 7), and (3) FWP and FWP% of, respectively, 15 and 11 parameters (0.91≤ICC≤0.96, 8.4%≤|SEM%|≤13.6%) (Table 8).

Examining the gender comparison results of the reference spatiotemporal gait parameters reveals that men maintain a higher swing duration and stride length across the three walking conditions. At the PW condition, men have a 5% increase in GS, which is associated with an 8% increase in SL and a 3% decrease in Cad, compared to women. These observations are supported by several laboratory-based gait studies in healthy adults aged 40–65 years (e.g., [15,16]). It is, however, noteworthy that these reference values are more consistent with those obtained in relatively long walkways (which is the case here, i.e., 30 m) (e.g., [9]: 40 m; [10]: 20 m) than those obtained in short walkways (e.g., [17]: 5.5 m). For example, our GS values for men are 6% to 15% higher than those reported in [17].

At the DTW condition with serial sevens subtractions, men exhibit a 4% increase in Sr, a 3% increase in Sw, a 9% increase in SL, and a 4% decrease in Cad compared to women, while their GSs remain similar (*p* > 0.05). These gender-based DTW findings are not readily available in previous studies. Additionally, our DTW results align with those reported in [18]. The reported values in [18], compared to those obtained in this study, are as follows: Sr: 1.1 (0.1) s vs. 1.090 (0.124) s; Sa%: 62.3 (1.2)% vs. 62.87 (1.6)%; SL: 1.4 (0.2) m vs. 1.487 (0.158) m; and GS: 1.3 (0.2) m/s vs. 1.383 (0.223) m/s.

At the FW condition, men show a 10% increase in their GS compared to women. This increase is associated with higher SL, Sw, and Sw% values. These observations are consistent with those reported in [19] for healthy adults aged 40–65 years. Contrary to this previous study, we found, however, that (1) men had a lower Sa% and DS%, while having a higher SL_n1_ and SL_n2_, and (2) higher SL and GS values in both genders. In this previous study, barefoot walking is considered, which can lead to differences in gait parameter values compared to those obtained using personal shoes [20], as used in our study.

The gait of the healthy participants is expected to be symmetric, which is reflected in the SI4 reference values that are all close to one. Interestingly, these values show a clear gender effect of DS, St, DS%, and St% on the SI4 at PW, and of DS and DS% at DTW and FW. In [10], no gender effect is found on the SI4 of St at PW. This difference with our results may be due to the use of a different symmetry index. The latter is calculated in [10], using a formula equivalent to the one used for SI1. We demonstrate, however, that SI1 shows a moderate relative (ICC [95% CI] = 0.53 [0.38,0.66]) and poor absolute (SEM% = 29.5%, and MDC% = 81.9%) reliability, which may limit the usefulness of this symmetry outcome, at least in experimental conditions similar to the ones of our study.

Among all the gait parameters, SL and DS% are the key parameters for which FWP% reference values present a gender difference. Men mainly increased their SL and decreased their DS% to increase their GS from PW to FW. To the best of our knowledge, these FWP and FWP% reference values and gender comparisons are not readily available in previous studies. Some studies report some of these values, but mainly for older adults aged at least 65 years (e.g., [40]). Based on these findings, one may consider the FWP and FWP% as reliable clinical features in fast walking studies. Further work should focus on investigating whether these features are associated with mechanisms of abnormal gait.

### 4.4. Advancements in IMU-Based Gait Analysis, Study Limitations, and Directions for Future Research

During the development of the hardware and signal-processing algorithms of our system, we ensured that they complied with the transparency requirements necessary for a wearable system intended to derive medical-grade features. Transparency, related to traceability and explainability [43], is achieved by (1) validating this system, and (2) engaging in regular discussions with medical practitioners and doctors to (a) better define the features needed for their specific needs, and (b) provide detailed information on all steps involved in feature extraction, including fundamental gait event identification and pre- and post-processing methods. To enhance explainability, we directly apply the signal-processing algorithms on raw data, enabling the easy visualization and verification of the results, which mitigates the algorithm “black box” issue in the context of using an IMU-based method for medical applications.

The current hardware configuration ensures perfect time synchronization in inertial data recording, enabling the signal-processing algorithms to accurately quantify gait parameters that require synchronized data from both feet, such as double-support and step durations, as well as the symmetry and fast walking performance indices associated with these durations. The results also demonstrate the hardware system’s ability to handle the recording of many consecutive strides, which could result in the reliable extraction of variability parameters. Additionally, our approach relies on versatile signal-processing algorithms, which provides a real advantage in quantifying other reference gait parameters such as the stride width, toe clearance, and durations of the sub-phases that refine the stance and swing phases [27]. The algorithms could be adapted to consider abnormal gait patterns, such as foot-drop after stroke [44] or the freezing of gait in Parkinson’s disease [45]. Overall, the proposed method shows significant potential in rehabilitation, geriatrics, orthopedics, and sports.

One limitation of this paper is its almost exclusive focus on assessing the intra-session reliability of the gait parameters. Another limitation is the lack of a formal evaluation of the DTW outcomes. Future studies should examine both the intra- and inter-session reliability in patient groups. We will explore establishing a more generalized reference database by incorporating additional anthropometric factors such as fitness level, BMI, and ethnicity. By leveraging both the (raw) signals from the toe IMUs and the versatility of the signal-processing algorithms, we will also extract the sub-phase durations, which refine the stance and swing phases [27], to assess their reliability.

## 5. Conclusions

Using a validated system using one IMU module on each of the two heels of each subject, we quantified clinically meaningful gait parameters including spatiotemporal gait parameters, symmetry, the dual-task cost, and fast walking performance indices, in 101 healthy adults (55 men and 46 women) aged 40–65 years, under preferred, dual-task, and fast walking conditions. We analyzed these gait parameters (1) to evaluate the level of their relative and absolute intra-session reliability, and (2) to establish a new database of reference values for the parameters that show good to excellent reliability. The results show that this database offers accurate and reliable reference values related to gender, leg length, and body height. The results are consistent with previous studies, while also offering new gait parameter information seldom reported in the literature, such as gender-based FWP comparisons and the symmetry ratios at serial sevens DTW and FW. The proposed IMU-based system offers significant advantages, including its transparency and ability to be used in clinical environments. Overall, the results obtained support the use of this system in a variety of medical applications, such as prosthetic evaluation, fall risk assessment, and neurological rehabilitation (e.g., Parkinson’s Disease, stroke).

## Figures and Tables

**Table 1:** Mean (standard deviation (SD)) age, anthropometric data of the participants, and gender comparisons.

	W (n = 46)		M (n = 55)		M & W (n = 101)		W vs. M
	Mean (SD)	min–max	Mean (SD)	min–max	Mean (SD)	min–max	*p*
Age [years]	51.1 (5.4)	40.8–63.7	52.7 (5.8)	40.4–65.9	52.0 (5.7)	40.4–65.9	0.167
Height [cm]	163.2 (5.1)	150.9–172.6	176.7 (6.4)	161.0–194.0	170.6 (8.9)	150.9–194.0	**<0.00001**
Weight [kg]	66.9 (10.8)	49.0–93.0	84.5 (12.6)	57.0–126.0	76.5 (14.7)	49.0–126.0	**<0.00001**
BMI [kg/m^2^]	24.4 (4.2)	19.1–35.0	26.3 (3.5)	18.7–37.6	25.4 (3.9)	18.7–37.6	**0.0084**

W: women; M: men; BMI: Body mass index; *p*: *p*-value (for details on its calculation, refer to Section 2.4).

**Table 2:** Extraction of the individual values of spatiotemporal gait parameters from consecutive and overlapping left gait cycles *i* and right gait cycles *j*, as illustrated in Figure 1. These parameters are *stride time* (Sr), *stance time* (Sa), *swing time* (Sw), *double-support time* (DS), *step time* (St), *cadence* (Cad), *stride length* (SL), and *gait speed* (GS). Sa%, Sw%, DS%, and St% are Sa, Sw, DS, and St as percentages of Sr, respectively.

Gait Parameters	Individual Values from Left Gait Cycles i	Individual Values from Right Gait Cycles j
Sr [s]	${{Sr}_{left}\left(i\right)=HS}_{left}\left(i+1\right)-{HS}_{left}\left(i\right)$	${{Sr}_{right}\left(j\right)=HS}_{right}\left(j+1\right)-{HS}_{right}\left(j\right)$
Sa [s]	${{Sa}_{left}\left(i\right)=TO}_{left}\left(i\right)-{HS}_{left}\left(i\right)$	${Sa}_{right}\left(j\right)={TO}_{right}\left(j\right)-{HS}_{right}\left(j\right)$
Sw [s]	${Sw}_{left}\left(i\right)={HS}_{left}\left(i+1\right)-{TO}_{left}\left(i\right)$	${{Sw}_{right}\left(j\right)=HS}_{right}\left(j+1\right)-{TO}_{right}\left(j\right)$
DS [s]	${{DS}_{left}\left(i\right)=TO}_{left}\left(i\right)-{HS}_{right}\left(j+1\right)$	${DS}_{right}\left(j\right)={TO}_{right}\left(j\right)-{HS}_{left}\left(i\right)$
St [s]	${{St}_{left}\left(i\right)=HS}_{left}\left(i\right)-{HS}_{right}\left(j\right)$	${{St}_{right}\left(j\right)=HS}_{right}\left(j+1\right)-{HS}_{left}\left(i\right)$
Cad [strides/s]	${Cad}_{left}\left(i\right)=1/{Sr}_{left}\left(i\right)$	${Cad}_{right}\left(j\right)=1/{Sr}_{right}\left(j\right)$
SL [m]	${SL}_{left}\left(i\right)\;$^(^*^)^	${SL}_{right}\left(j\right)\;$^(^*^)^
GS [m/s]	${GS}_{left}\left(i\right)={SL}_{left}\left(i\right)/{Sr}_{left}\left(i\right)$	${GS}_{right}\left(j\right)={SL}_{right}\left(j\right)/{Sr}_{right}\left(j\right)$
Sa% [%]	${Sa}_{left}\%\left(i\right)={100Sa}_{left}\left(i\right)/{Sr}_{left}\left(i\right)$	${Sa}_{right}\%\left(j\right)={100Sa}_{right}\left(j\right)/{Sr}_{right}\left(j\right)$
Sw% [%]	${Sw}_{left}\%\left(i\right)={100Sw}_{left}\left(i\right)/{Sr}_{left}\left(i\right)$	${Sw}_{right}\%\left(j\right)={100Sw}_{right}\left(j\right)/{Sr}_{right}\left(j\right)$
DS% [%]	${DS}_{left}\%\left(i\right)={100DS}_{left}\left(i\right)/{Sr}_{left}\left(i\right)$	${DS}_{right}\%\left(j\right)={100DS}_{right}\left(j\right)/{Sr}_{right}\left(j\right)$
St% [%]	${St}_{left}\%\left(i\right)={100St}_{left}\left(i\right)/{Sr}_{left}\left(i\right)$	${St}_{right}\%\left(j\right)={100St}_{right}\left(j\right)/{Sr}_{right}\left(j\right)$

^(^*^)^$\;{SL}_{left}\left(i\right)\;$ and ${SL}_{right}\left(j\right)$ are calculated using the method from [22].

**Table 3:** Intra-session reliability results of spatiotemporal gait parameters in 55 men and 46 women, all healthy and aged 40−65, in normal, dual-task, and fast walking conditions. These parameters include *stride time* (Sr) [s], *stance time* (Sa) [s], *swing time* (Sw) [s], *double-support time* (DS) [s], *step time* (St) [s], *stride length* (SL) [m], *gait speed* (GS) [m/s], and *cadence* (Cad) [strides/s]. Sa%, Sw%, and DS% are Sa, Sw, and DS as percentages [%] of Sr. SL_n1_ [dimensionless] and GS_n1_ [s^−1^] are SL and GS normalized to leg length, and SL_n2_ [dimensionless] and GS_n2_ [s^−1^] are SL and GS normalized to body height.

		Trial1: Mean (SD)	Trial2: Mean (SD)	ICC(2.1) [95% CI]	LOA [95% CI]	SEM (SEM%)	MDC (MDC%)	*p*
Preferred walking	Sr	1.040 (0.054)	1.043 (0.054)	0.97 [0.96, 0.98]	[−0.020, 0.026]	0.009 (0.8)	0.024 (2.3)	0.59
	Sa	0.650 (0.043)	0.653 (0.041)	0.97 [0.96, 0.98]	[−0.016, 0.021]	0.007 (1.0)	0.019 (2.9)	0.59
	Sw	0.389 (0.021)	0.390 (0.021)	0.98 [0.97, 0.99]	[−0.007, 0.009]	0.003 (0.7)	0.008 (2.1)	0.86
	DS	0.131 (0.020)	0.132 (0.019)	0.98 [0.96, 0.99]	[−0.007, 0.009]	0.003 (2.2)	0.008 (6.1)	0.66
	St	0.520 (0.027)	0.521 (0.027)	0.97 [0.96, 0.98]	[−0.010, 0.013]	0.004 (0.8)	0.012 (2.3)	0.62
	SL	1.529 (0.142)	1.526 (0.138)	0.99 [0.99, 0.99]	[−0.042, 0.035]	0.014 (0.9)	0.039 (2.5)	0.86
	GS	1.475 (0.153)	1.468 (0.145)	0.98 [0.97, 0.98]	[−0.069, 0.055]	0.023 (1.5)	0.063 (4.3)	0.74
	Cad	0.965 (0.049)	0.962 (0.048)	0.97 [0.96, 0.98]	[−0.024, 0.018]	0.008 (0.8)	0.022 (2.3)	0.68
	Sa%	62.52 (1.54)	62.58 (1.50)	0.99 [0.98, 0.99]	[−0.45, 0.56]	0.19 (0.3)	0.52 (0.8)	0.79
	Sw%	37.48 (1.54)	37.42 (1.50)	0.99 [0.98, 0.99]	[−0.56, 0.45]	0.19 (0.5)	0.52 (1.4)	0.79
	DS%	12.50 (1.54)	12.57 (1.49)	0.98 [0.98, 0.99]	[−0.43, 0.58]	0.19 (1.5)	0.52 (4.14)	0.74
	SL_n1_	1.723 (0.143)	1.719 (0.139)	0.99 [0.98, 0.99]	[−0.046, 0.038]	0.015 (0.9)	0.042 (2.5)	0.84
	GS_n1_	1.663 (0.172)	1.655 (0.162)	0.98 [0.97, 0.98]	[−0.077, 0.061]	0.025 (1.5)	0.070 (4.2)	0.74
	SL_n2_	0.896 (0.069)	0.894 (0.066)	0.99 [0.98, 0.99]	[−0.024, 0.020]	0.008 (0.9)	0.022 (2.5)	0.83
	GS_n2_	0.865 (0.086)	0.861 (0.082)	0.98 [0.96, 0.98]	[−0.040, 0.032]	0.013 (1.5)	0.037 (4.2)	0.74
Dual-task walking	Sr	1.090 (0.134)	1.088 (0.116)	0.95 [0.93, 0.97]	[−0.080, 0.076]	0.028 (2.6)	0.078 (7.1)	0.79
	Sa	0.686 (0.090)	0.685 (0.078)	0.95 [0.92, 0.96]	[−0.055, 0.053]	0.019 (2.8)	0.053 (7.8)	0.75
	Sw	0.405 (0.048)	0.403 (0.040)	0.96 [0.94, 0.97]	[−0.027, 0.023]	0.009 (2.2)	0.025 (6.2)	0.99
	DS	0.140 (0.028)	0.141 (0.026)	0.95 [0.92, 0.96]	[−0.017, 0.018]	0.006 (4.4)	0.017 (12.1)	0.87
	St	0.545 (0.066)	0.544 (0.058)	0.95 [0.93, 0.97]	[−0.038, 0.036]	0.013 (2.4)	0.036 (6.7)	0.81
	SL	1.490 (0.160)	1.483 (0.158)	0.97 [0.96, 0.98]	[−0.081, 0.067]	0.027 (1.8)	0.075 (5.0)	0.75
	GS	1.386 (0.231)	1.381 (0.219)	0.97 [0.95, 0.98]	[−0.119, 0.108]	0.041 (3.0)	0.113 (8.2)	0.82
	Cad	0.929 (0.097)	0.929 (0.089)	0.96 [0.95, 0.98]	[−0.049, 0.050]	0.018 (2.0)	0.049 (5.3)	0.84
	Sa%	62.82 (1.62)	62.91 (1.66)	0.96 [0.94, 0.97]	[−0.81, 0.98]	0.32 (0.5)	0.90 (1.4)	0.72
	Sw%	37.18 (1.62)	37.09 (1.66)	0.96 [0.94, 0.97]	[−0.98, 0.81]	0.32 (0.9)	0.90 (2.4)	0.72
	DS%	12.79 (1.61)	12.88 (1.65)	0.96 [0.94, 0.97]	[−0.80, 0.98]	0.33 (2.5)	0.90 (7.0)	0.70
	SL_n1_	1.678 (0.152)	1.670 (0.153)	0.96 [0.94, 0.97]	[−0.092, 0.076]	0.031 (1.8)	0.085 (5.1)	0.72
	GS_n1_	1.562 (0.249)	1.556 (0.236)	0.96 [0.94, 0.97]	[−0.137, 0.124]	0.047 (3.0)	0.130 (8.4)	0.81
	SL_n2_	0.873 (0.077)	0.869 (0.076)	0.96 [0.94, 0.97]	[−0.048, 0.040]	0.016 (1.8)	0.044 (5.1)	0.70
	GS_n2_	0.813 (0.129)	0.810 (0.122)	0.96 [0.94, 0.97]	[−0.071, 0.065]	0.024 (3.0)	0.068 (8.4)	0.83
Fast walking	Sr	0.882 (0.065)	0.890 (0.065)	0.96 [0.92, 0.97]	[−0.027, 0.043]	0.014 (1.5)	0.038 (4.3)	0.39
	Sa	0.529 (0.043)	0.535 (0.044)	0.95 [0.89, 0.97]	[−0.018, 0.031]	0.010 (1.8)	0.027 (5.1)	0.28
	Sw	0.354 (0.026)	0.355 (0.026)	0.97 [0.96, 0.98]	[−0.011, 0.013]	0.004 (1.3)	0.012 (3.5)	0.79
	DS	0.087 (0.015)	0.090 (0.016)	0.94 [0.85, 0.97]	[−0.006, 0.012]	0.004 (4.4)	0.011 (12.1)	0.20
	St	0.441 (0.032)	0.445 (0.032)	0.96 [0.92, 0.97]	[−0.013, 0.021]	0.007 (1.5)	0.019 (4.3)	0.38
	SL	1.789 (0.173)	1.765 (0.166)	0.97 [0.90, 0.99]	[−0.091, 0.043]	0.029 (1.6)	0.081 (4.6)	0.32
	GS	2.039 (0.253)	1.995 (0.235)	0.95 [0.85, 0.97]	[−0.176, 0.087]	0.056 (2.8)	0.156 (7.7)	0.19
	Cad	1.140 (0.088)	1.131 (0.086)	0.95 [0.92, 0.97]	[−0.060, 0.040]	0.019 (1.7)	0.053 (4.7)	0.42
	Sa%	59.95 (1.36)	60.19 (1.34)	0.92 [0.84, 0.95]	[−0.75, 1.23]	0.39 (0.6)	1.08 (1.8)	0.21
	Sw%	40.05 (1.36)	39.81 (1.34)	0.92 [0.84, 0.95]	[−1.23, 0.75]	0.39 (1.0)	1.08 (2.7)	0.21
	DS%	9.91 (1.37)	10.17 (1.34)	0.91 [0.82, 0.95]	[−0.77, 1.30]	0.41 (4.1)	1.14 (11.4)	0.17
	SL_n1_	2.013 (0.176)	1.985 (0.166)	0.96 [0.88, 0.98]	[−0.107, 0.052]	0.034 (1.7)	0.095 (4.8)	0.25
	GS_n1_	2.297 (0.294)	2.246 (0.264)	0.95 [0.85, 0.97]	[−0.203, 0.101]	0.065 (2.9)	0.180 (7.9)	0.22
	SL_n2_	1.046 (0.077)	1.032 (0.072)	0.94 [0.83, 0.97]	[−0.055, 0.027]	0.018 (1.7)	0.049 (4.7)	0.18
	GS_n2_	1.194 (0.132)	1.167 (0.116)	0.93 [0.80, 0.97]	[−0.105, 0.051]	0.033 (2.8)	0.093 (7.8)	0.18

**Table 4:** Intra-session reliability results for symmetry index SI4 in 55 men and 46 women, all healthy and aged 40–65, at preferred, dual-task, and fast walking speeds. The index values are obtained by applying Formula 4 to *stance time* (Sa), *swing time* (Sw), *double-support time* (DS), *step time* (St), their respective percentages (Sa%, Sw%, DS%, and St%) of stride time, *stride length* (SL), and *gait speed* (GS).

		Trial1: Mean (SD)	Trial2: Mean (SD)	ICC(2.1) [95% CI]	LOA [95% CI]	SEM (SEM%)	MDC (MDC%)	*p*
Preferred walking	Sa	1.000 (0.012)	1.002 (0.012)	0.82 [0.74, 0.88]	[−0.012, 0.015]	0.005 (0.5)	0.014 (1.4)	0.38
	Sw	1.001 (0.020)	0.998 (0.019)	0.81 [0.73, 0.87]	[−0.026, 0.021]	0.009 (0.9)	0.024 (2.4)	0.36
	DS	0.984 (0.064)	0.984 (0.059)	0.91 [0.87, 0.94]	[−0.051, 0.052]	0.018 (1.9)	0.051 (5.2)	0.97
	St	0.996 (0.021)	0.994 (0.020)	0.80 [0.72, 0.86]	[−0.028, 0.022]	0.009 (0.9)	0.025 (2.5)	0.35
	SL	1.007 (0.031)	1.009 (0.031)	0.93 [0.89, 0.95]	[−0.021, 0.025]	0.008 (0.8)	0.023 (2.3)	0.64
	GS	1.008 (0.031)	1.010 (0.031)	0.94 [0.91, 0.96]	[−0.020, 0.024]	0.008 (0.8)	0.022 (2.1)	0.60
	Sa%	1.000 (0.012)	1.002 (0.012)	0.83 [0.75, 0.88]	[−0.011, 0.015]	0.005 (0.5)	0.014 (1.4)	0.28
	Sw%	1.001 (0.020)	0.998 (0.020)	0.84 [0.76, 0.89]	[−0.025, 0.019]	0.008 (0.8)	0.022 (2.2)	0.29
	DS%	0.983 (0.064)	0.985 (0.060)	0.90 [0.86, 0.93]	[−0.052, 0.056]	0.019 (2.0)	0.054 (5.5)	0.82
	St%	0.996 (0.021)	0.994 (0.020)	0.81 [0.73, 0.87]	[−0.027, 0.022]	0.009 (1.0)	0.025 (2.5)	0.35
Dual-task walking	Sa	1.000 (0.015)	1.002 (0.014)	0.71 [0.59, 0.79]	[−0.020, 0.024]	0.008 (0.8)	0.022 (2.2)	0.30
	Sw	1.002 (0.024)	0.998 (0.024)	0.78 [0.69, 0.85]	[−0.035, 0.027]	0.011 (1.1)	0.031 (3.1)	0.29
	DS	0.992 (0.060)	0.982 (0.064)	0.84 [0.76, 0.89]	[−0.077, 0.057]	0.025 (2.5)	0.069 (7.0)	0.26
	St	0.997 (0.026)	0.992 (0.025)	0.73 [0.61, 0.81]	[−0.041, 0.031]	0.013 (1.3)	0.037 (3.7)	0.15
	SL	1.009 (0.038)	1.008 (0.038)	0.94 [0.92, 0.96]	[−0.026, 0.025]	0.009 (0.9)	0.025 (2.5)	0.85
	GS	1.009 (0.038)	1.009 (0.039)	0.93 [0.90, 0.95]	[−0.027, 0.028]	0.010 (1.0)	0.028 (2.7)	0.95
	Sa%	1.000 (0.014)	1.002 (0.014)	0.75 [0.65, 0.83]	[−0.018, 0.021]	0.007 (0.7)	0.020 (2.0)	0.38
	Sw%	1.001 (0.025)	0.998 (0.025)	0.77 [0.68, 0.84]	[−0.036, 0.030]	0.012 (1.2)	0.033 (3.3)	0.41
	DS%	0.992 (0.060)	0.981 (0.064)	0.84 [0.76, 0.89]	[−0.078, 0.057]	0.025 (2.6)	0.070 (7.1)	0.24
	St%	0.997 (0.025)	0.993 (0.025)	0.79 [0.70, 0.86]	[−0.035, 0.026]	0.011 (1.1)	0.031 (3.1)	0.20
Fast walking	Sa	1.002 (0.015)	1.003 (0.015)	0.79 [0.71, 0.86]	[−0.017, 0.021]	0.007 (0.7)	0.019 (1.9)	0.45
	Sw	1.000 (0.022)	0.997 (0.023)	0.82 [0.74, 0.88]	[−0.029, 0.022]	0.009 (0.9)	0.026 (2.6)	0.30
	DS	0.975 (0.085)	0.975 (0.078)	0.73 [0.62, 0.81]	[−0.120, 0.119]	0.043 (4.4)	0.118 (12.1)	0.76
	St	0.994 (0.021)	0.990 (0.020)	0.59 [0.45, 0.71]	[−0.040, 0.031]	0.013 (1.3)	0.037 (3.7)	0.12
	SL	1.005 (0.029)	1.006 (0.029)	0.90 [0.86, 0.93]	[−0.025, 0.026]	0.009 (1.0)	0.025 (2.5)	0.92
	GS	1.006 (0.030)	1.006 (0.029)	0.89 [0.85, 0.93]	[−0.026, 0.027]	0.010 (0.9)	0.026 (2.6)	0.90
	Sa%	1.001 (0.015)	1.003 (0.015)	0.81 [0.73, 0.87]	[−0.016, 0.019]	0.006 (0.6)	0.018 (1.8)	0.36
	Sw%	0.999 (0.022)	0.997 (0.022)	0.81 [0.74, 0.87]	[−0.029, 0.024]	0.010 (0.9)	0.026 (2.6)	0.45
	DS%	0.975 (0.086)	0.975 (0.079)	0.72 [0.61, 0.80]	[−0.122, 0.121]	0.044 (4.5)	0.121 (12.4)	0.97
	St%	0.993 (0.021)	0.991 (0.020)	0.65 [0.52, 0.75]	[−0.036, 0.031]	0.012 (1.2)	0.034 (3.4)	0.32

**Table 5:** Intra-session reliability results of fast walking performance indices, FWP and FWP%, in 55 men and 46 women, all healthy and aged 40–65. These indices are obtained for *stride time* (Sr), *stance time* (Sa), *swing time* (Sw), *double-support time* (DS), their respective percentages (Sa%, Sw%, and DS%) of Sr, *step time* (St), *stride length* (SL), *gait speed* (GS), *cadence* (Cad), SL, and GS normalized to leg length (SL_n1_ and GS_n1_) and body height (SL_n2_ and GS_n2_).

		Trial1: Mean (SD)	Trial2: Mean (SD)	ICC(2.1) [95% CI]	LOA [95% CI]	SEM (SEM%)	MDC (MDC%)	*p*
Fast walking performance index FWP	Sr	−0.157 (0.073)	−0.153 (0.071)	0.96 [0.94, 0.97]	[−0.035, 0.044]	0.01 (−9.3)	0.04 (−25.8)	0.67
	Sa	−0.121 (0.054)	−0.118 (0.053)	0.96 [0.94, 0.97]	[−0.025, 0.032]	0.01 (−8.7)	0.03 (−24.2)	0.53
	Sw	−0.035 (0.021)	−0.035 (0.021)	0.95 [0.92, 0.96]	[−0.013, 0.014]	0.00 (−13.6)	0.01 (−37.7)	0.84
	DS	−0.043 (0.019)	−0.042 (0.019)	0.96 [0.93, 0.97]	[−0.009, 0.012]	0.00 (−9.5)	0.01 (−26.4)	0.60
	St	−0.079 (0.036)	−0.076 (0.036)	0.96 [0.94, 0.97]	[−0.018, 0.022]	0.01 (−9.4)	0.02 (−25.9)	0.62
	SL	0.258 (0.116)	0.239 (0.112)	0.94 [0.86, 0.96]	[−0.091, 0.053]	0.03 (11.6)	0.08 (32.3)	0.22
	GS	0.563 (0.246)	0.528 (0.235)	0.95 [0.89, 0.97]	[−0.171, 0.100]	0.05 (10.0)	0.15 (27.6)	0.23
	Cad	0.176 (0.091)	0.169 (0.089)	0.95 [0.93, 0.97]	[−0.059, 0.046]	0.02 (11.2)	0.05 (31.1)	0.51
	Sa%	−2.637 (1.252)	−2.498 (1.231)	0.94 [0.90, 0.96]	[−0.697, 0.974]	0.31 (−12.2)	0.87 (−33.9)	0.37
	Sw%	2.637 (1.252)	2.498 (1.231)	0.94 [0.90, 0.96]	[−0.974, 0.697]	0.31 (12.2)	0.87 (33.9)	0.37
	DS%	−2.650 (1.244)	−2.499 (1.229)	0.93 [0.89, 0.96]	[−0.684, 0.986]	0.32 (−12.3)	0.88 (−34.1)	0.36
	SL_n1_	0.291 (0.131)	0.269 (0.126)	0.94 [0.87, 0.97]	[−0.101, 0.058]	0.03 (11.5)	0.09 (31.9)	0.21
	GS_n1_	0.637 (0.283)	0.596 (0.270)	0.95 [0.90, 0.97]	[−0.193, 0.112]	0.06 (10.0)	0.17 (27.6)	0.25
	SL_n2_	0.151 (0.065)	0.139 (0.063)	0.93 [0.85, 0.96]	[−0.053, 0.030]	0.02 (12.0)	0.05 (32.2)	0.22
	GS_n2_	0.329 (0.138)	0.308 (0.132)	0.94 [0.88, 0.97]	[−0.100, 0.058]	0.03 (10.0)	0.09 (27.6)	0.23
Fast walking performance index FWP%	Sr	−14.987 (6.452)	−14.542 (6.347)	0.96 [0.93, 0.97]	[−3.223, 4.114]	1.35 (−9.1)	3.74 (−25.3)	0.61
	Sa	−18.443 (7.233)	−17.831 (7.196)	0.95 [0.93, 0.97]	[−3.491, 4.715]	1.53 (−8.4)	4.24 (−23.4)	0.44
	Sw	−9.066 (5.335)	−8.962 (5.262)	0.95 [0.92, 0.96]	[−3.311, 3.519]	1.23 (−13.6)	3.40 (−37.7)	0.81
	DS	−32.296 (10.533)	−30.947 (10.605)	0.93 [0.89, 0.96]	[−5.988, 8.685]	2.79 (−8.8)	7.73 (−24.4)	0.36
	St	−14.999 (6.404)	−14.525 (6.334)	0.95 [0.93, 0.97]	[−3.197, 4.145]	1.36 (−9.2)	3.76 (−25.5)	0.58
	SL	17.103 (8.234)	15.870 (7.909)	0.94 [0.88, 0.97]	[−6.121, 3.655]	1.95 (11.8)	5.40 (32.8)	0.24
	GS	39.097 (19.651)	36.824 (18.985)	0.96 [0.92, 0.98]	[−12.204, 7.658]	3.89 (10.3)	10.80 (28.4)	0.31
	Cad	18.414 (10.065)	17.761 (9.866)	0.96 [0.94, 0.97]	[−6.177, 4.872]	2.03 (11.2)	5.63 (31.1)	0.60
	Sa%	−4.195 (1.914)	−3.971 (1.882)	0.93 [0.89, 0.96]	[−1.094, 1.541]	0.50 (−12.2)	1.38 (−33.7)	0.35
	Sw%	7.118 (3.633)	6.751 (3.570)	0.94 [0.91, 0.96]	[−2.661, 1.928]	0.86 (12.4)	2.38 (34.4)	0.40
	DS%	−20.928 (8.252)	−19.616 (8.207)	0.91 [0.85, 0.94]	[−5.120, 7.744]	2.48 (−12.2)	6.86 (−33.8)	0.27

**Table 6:** Gender effect on reference values for spatiotemporal gait parameters at preferred, dual-task, and fast walking speeds (trials 1 and 2). Parameters include *stride time* (Sr) [s], *stance time* (Sa) [s], *swing time* (Sw) [s], *double-support time* (DS) [s], *step time* (St) [s], their percentages (Sa%, Sw%, DS%, and St%) of Sr, *stride length* (SL) [m], *gait speed* (GS) [m/s], and their values normalized to leg length (SL_n1_ [dimensionless] and GS_n1_ [s^−1^]) and body height (SL_n2_ [dimensionless] and GS_n2_ [s^−1^]), and *cadence* (Cad) [strides/s].

		Women (n = 46)		Men (n = 55)		Men and Women (n = 101)		*p*
		Mean (SD)	95% CI	Mean (SD)	95% CI	Mean (SD)	95% CI	
Preferred walking	Sr	1.024 (0.043)	[1.011, 1.037]	1.056 (0.057)	[1.041, 1.071]	1.041 (0.054)	[1.030, 1.052]	**0.003**
	Sa	0.643 (0.037)	[0.632, 0.654]	0.658 (0.044)	[0.646, 0.670]	0.652 (0.042)	[0.644, 0.660]	0.069
	Sw	0.380 (0.017)	[0.375, 0.385]	0.397 (0.021)	[0.391, 0.403]	0.390 (0.021)	[0.386, 0.394]	**0.00002**
	DS	0.132 (0.020)	[0.126, 0.138]	0.131 (0.019)	[0.126, 0.136]	0.131 (0.019)	[0.127, 0.135]	0.784
	St	0.512 (0.021)	[0.506, 0.518]	0.528 (0.029)	[0.520, 0.536]	0.521 (0.027)	[0.516, 0.526]	**0.003**
	SL	1.461 (0.128)	[1.423, 1.499]	1.583 (0.125)	[1.549, 1.617]	1.527 (0.139)	[1.500, 1.554]	**0.00001**
	GS	1.432 (0.149)	[1.388, 1.476]	1.504 (0.141)	[1.466, 1.542]	1.471 (0.148)	[1.442, 1.500]	**0.014**
	Cad	0.979 (0.042)	[0.967, 0.991]	0.950 (0.050)	[0.936, 0.964]	0.963 (0.048)	[0.954, 0.972]	**0.003**
	Sa%	62.81 (1.63)	[62.33, 63.30]	62.33 (1.39)	[61.95, 62.70]	62.55 (1.52)	[62.27, 62.82]	0.110
	Sw%	37.19 (1.63)	[36.70, 37.67]	37.67 (1.39)	[37.30, 38.05]	37.45 (1.52)	[37.18, 37.73]	0.110
	DS%	12.79 (1.62)	[12.31, 13.27]	12.32 (1.39)	[11.94, 12.69]	12.53 (1.51)	[12.26, 12.81]	0.116
	SL_n1_	1.695 (0.127)	[1.657, 1.733]	1.743 (0.148)	[1.703, 1.783]	1.721 (0.140)	[1.693, 1.749]	0.082
	GS_n1_	1.661 (0.160)	[1.613, 1.709]	1.657 (0.172)	[1.611, 1.703]	1.659 (0.166)	[1.626, 1.692]	0.924
	SL_n2_	0.895 (0.063)	[0.876, 0.914]	0.896 (0.071)	[0.877, 0.915]	0.895 (0.067)	[0.882, 0.908]	0.924
	GS_s_	0.877 (0.079)	[0.854, 0.9]	0.852 (0.086)	[0.829, 0.875]	0.863 (0.084)	[0.846, 0.880]	0.144
Dual-task walking	Sr	1.068 (0.139)	[1.027, 1.109]	1.108 (0.108)	[1.079, 1.137]	1.090 (0.124)	[1.066, 1.114]	**0.00133**
	Sa	0.675 (0.092)	[0.648, 0.702]	0.694 (0.076)	[0.673, 0.715]	0.685 (0.083)	[0.669, 0.701]	0.269
	Sw	0.392 (0.050)	[0.377, 0.407]	0.414 (0.036)	[0.404, 0.424]	0.404 (0.044)	[0.395, 0.413]	**0.00002**
	DS	0.141 (0.028)	[0.133, 0.149]	0.140 (0.025)	[0.133, 0.147]	0.141 (0.026)	[0.136, 0.146]	0.792
	St	0.534 (0.069)	[0.514, 0.554]	0.554 (0.054)	[0.539, 0.569]	0.545 (0.061)	[0.533, 0.557]	0.101
	SL	1.420 (0.140)	[1.378, 1.462]	1.543 (0.151)	[1.502, 1.584]	1.487 (0.158)	[1.456, 1.518]	**0.00005**
	GS	1.350 (0.206)	[1.289, 1.411]	1.411 (0.235)	[1.347, 1.475]	1.383 (0.223)	[1.339, 1.427]	0.168
	Cad	0.949 (0.096)	[0.92, 0.978]	0.912 (0.086)	[0.889, 0.935]	0.929 (0.092)	[0.911, 0.947]	**0.04428**
	Sa%	63.20 (1.68)	[62.70, 63.70]	62.59 (1.54)	[62.17, 63.01]	62.87 (1.63)	[62.56, 63.17]	0.062
	Sw%	36.80 (1.68)	[36.30, 37.30]	37.41 (1.54)	[36.99, 37.83]	37.13 (1.63)	[36.83, 37.44]	0.062
	DS%	13.18 (1.66)	[12.69, 13.68]	12.55 (1.53)	[12.14, 12.97]	12.84 (1.61)	[12.54, 13.14]	0.051
	SL_n1_	1.646 (0.139)	[1.605, 1.687]	1.698 (0.158)	[1.655, 1.741]	1.674 (0.151)	[1.644, 1.704]	0.083
	GS_n1_	1.566 (0.232)	[1.497, 1.635]	1.552 (0.248)	[1.485, 1.619]	1.559 (0.240)	[1.512, 1.606]	0.779
	SL_n2_	0.869 (0.071)	[0.848, 0.89]	0.873 (0.080)	[0.851, 0.895]	0.871 (0.076)	[0.856, 0.886]	0.797
	GS_n2_	0.826 (0.117)	[0.791, 0.861]	0.799 (0.130)	[0.764, 0.834]	0.811 (0.125)	[0.786, 0.836]	0.271
Fast walking	Sr	0.877 (0.057)	[0.860, 0.894]	0.894 (0.069)	[0.876, 0.913]	0.886 (0.064)	[0.873, 0.900]	0.175
	Sa	0.530 (0.040)	[0.518, 0.542]	0.533 (0.046)	[0.521, 0.546]	0.532 (0.043)	[0.523, 0.541]	0.735
	Sw	0.346 (0.023)	[0.340, 0.353]	0.361 (0.026)	[0.354, 0.368]	0.354 (0.026)	[0.349, 0.360]	**0.003**
	DS	0.092 (0.016)	[0.087, 0.097]	0.086 (0.014)	[0.082, 0.090]	0.089 (0.015)	[0.086, 0.092]	0.066
	St	0.438 (0.029)	[0.430, 0.447]	0.447 (0.034)	[0.438, 0.457]	0.443 (0.032)	[0.436, 0.450]	0.164
	SL	1.672 (0.147)	[1.629, 1.716]	1.864 (0.134)	[1.827, 1.900]	1.777 (0.169)	[1.750, 1.803]	**<0.00001**
	GS	1.915 (0.187)	[1.860, 1.971]	2.101 (0.251)	[2.033, 2.169]	2.016 (0.242)	[1.967, 2.066]	**0.00007**
	Cad	1.146 (0.079)	[1.123, 1.170]	1.126 (0.092)	[1.101, 1.151]	1.135 (0.086)	[1.117, 1.153]	0.216
	Sa%	60.50 (1.40)	[60.08, 60.91]	59.72 (1.16)	[59.40, 60.03]	60.07 (1.33)	[59.84, 60.30]	**0.00285**
	Sw%	39.50 (1.40)	[39.09, 39.92]	40.28 (1.16)	[39.97, 40.60]	39.93 (1.33)	[39.70, 40.16]	**0.00285**
	DS%	10.48 (1.40)	[10.06, 10.89]	9.69 (1.16)	[9.37, 10.00]	10.05 (1.33)	[9.82, 10.27]	**0.00254**
	SL_n1_	1.939 (0.147)	[1.896, 1.983]	2.054 (0.179)	[2.006, 2.103]	2.002 (0.174)	[1.967, 2.037]	**0.0007**
	GS_n1_	2.224 (0.228)	[2.156, 2.292]	2.318 (0.318)	[2.232, 2.404]	2.275 (0.283)	[2.212, 2.338]	0.097
	SL_n2_	1.024 (0.075)	[1.002, 1.046]	1.055 (0.073)	[1.035, 1.075]	1.041 (0.075)	[1.026, 1.055]	**0.039**
	GS_n2_	1.173 (0.106)	[1.142, 1.205]	1.189 (0.140)	[1.152, 1.227]	1.182 (0.126)	[1.154, 1.210]	0.519

**Table 7:** Effect of gender on reference values for symmetry ratio SI4 at preferred, dual-task, and fast walking speeds (trial 1 and 2). These values are obtained by applying Formula 4 to *stance time* (Sa), *swing time* (Sw), *double-support time* (DS), their percentages (Sa%, Sw%, and DS%) of Sr, *step time* (St), *stride length* (SL), and *gait speed* (GS).

		Women (n = 46)		Men (n = 55)		Men & Women (n = 101)		*p*
		Mean (SD)	95% CI	Mean (SD)	95% CI	Mean (SD)	95% CI	
Preferred walking	Sa	1.002 (0.013)	[0.998, 1.006]	1.001 (0.010)	[0.998, 1.004]	1.001 (0.011)	[0.999, 1.003]	0.740
	Sw	0.999 (0.021)	[0.993, 1.005]	1.000 (0.017)	[0.995, 1.005]	1.000 (0.019)	[0.996, 1.003]	0.803
	DS	0.967 (0.059)	[0.949, 0.985]	0.997 (0.058)	[0.981, 1.013]	0.984 (0.060)	[0.972, 0.996]	**0.012**
	St	0.990 (0.020)	[0.984, 0.996]	0.999 (0.019)	[0.994, 1.004]	0.995 (0.020)	[0.991, 0.999]	**0.013**
	SL	1.010 (0.026)	[1.002, 1.018]	1.007 (0.034)	[0.998, 1.016]	1.008 (0.030)	[1.002, 1.014]	0.581
	GS	1.011 (0.026)	[1.003, 1.019]	1.007 (0.034)	[0.998, 1.016]	1.009 (0.031)	[1.003, 1.015]	0.593
	Sa%	1.001 (0.012)	[0.997, 1.005]	1.001 (0.010)	[0.998, 1.004]	1.001 (0.011)	[0.999, 1.003]	0.739
	Sw%	0.998 (0.021)	[0.992, 1.004]	1.000 (0.017)	[0.995, 1.005]	0.999 (0.019)	[0.995, 1.003]	0.763
	DS%	0.968 (0.059)	[0.950, 0.986]	0.998 (0.059)	[0.982, 1.014]	0.984 (0.060)	[0.972, 0.996]	**0.014**
	St%	0.989 (0.020)	[0.983, 0.995]	0.999 (0.018)	[0.994, 1.004]	0.995 (0.020)	[0.991, 0.999]	**0.003**
Dual-task walking	Sa	1.001 (0.013)	[0.997, 1.005]	1.001 (0.014)	[0.997, 1.005]	1.001 (0.014)	[0.998, 1.004]	0.793
	Sw	1.001 (0.022)	[0.994, 1.008]	0.999 (0.023)	[0.993, 1.005]	1.000 (0.023)	[0.995, 1.004]	0.700
	DS	0.969 (0.058)	[0.952, 0.986]	1.002 (0.057)	[0.987, 1.017]	0.987 (0.060)	[0.975, 0.999]	**0.005**
	St	0.990 (0.023)	[0.983, 0.997]	0.999 (0.024)	[0.993, 1.005]	0.995 (0.024)	[0.990, 0.999]	0.060
	SL	1.010 (0.032)	[1.000, 1.020]	1.008 (0.042)	[0.997, 1.019]	1.009 (0.038)	[1.001, 1.016]	0.851
	GS	1.010 (0.032)	[1.000, 1.020]	1.008 (0.042)	[0.997, 1.019]	1.009 (0.038)	[1.001, 1.016]	0.814
	Sa%	1.001 (0.013)	[0.997, 1.005]	1.001 (0.014)	[0.997, 1.005]	1.001 (0.013)	[0.998, 1.004]	0.767
	Sw%	1.000 (0.023)	[0.993, 1.007]	0.999 (0.024)	[0.993, 1.005]	0.999 (0.023)	[0.995, 1.004]	0.711
	DS%	0.969 (0.059)	[0.951, 0.987]	1.001 (0.057)	[0.986, 1.016]	0.987 (0.060)	[0.975, 0.998]	**0.006**
	St%	0.991 (0.023)	[0.984, 0.998]	0.999 (0.023)	[0.993, 1.005]	0.995 (0.023)	[0.990, 1.000]	0.081
Fast walking	Sa	1.003 (0.015)	[0.999, 1.007]	1.002 (0.014)	[0.998, 1.006]	1.003 (0.014)	[1.000, 1.005]	0.804
	Sw	0.998 (0.023)	[0.991, 1.005]	0.998 (0.020)	[0.993, 1.003]	0.998 (0.021)	[0.994, 1.003]	0.991
	DS	0.954 (0.068)	[0.934, 0.974]	0.992 (0.078)	[0.971, 1.013]	0.975 (0.076)	[0.960, 0.990]	**0.012**
	St	1.006 (0.026)	[0.998, 1.014]	1.005 (0.030)	[0.997, 1.013]	1.005 (0.028)	[1.000, 1.011]	0.863
	SL	1.007 (0.027)	[0.999, 1.015]	1.005 (0.030)	[0.997, 1.013]	1.006 (0.029)	[1.000, 1.012]	0.738
	GS	1.002 (0.015)	[0.998, 1.006]	1.002 (0.014)	[0.998, 1.006]	1.002 (0.014)	[0.999, 1.005]	0.960
	Sa%	0.998 (0.023)	[0.991, 1.005]	0.998 (0.020)	[0.993, 1.003]	0.998 (0.021)	[0.994, 1.002]	0.970
	Sw%	0.954 (0.069)	[0.934, 0.974]	0.993 (0.078)	[0.972, 1.014]	0.975 (0.076)	[0.960, 0.990]	**0.009**

**Table 8:** Effect of gender on reference values for the fast walking performance indices FWP and FWP% from trials 1 and 2. These indices are obtained for *stride time* (Sr), *stance time* (Sa), *swing time* (Sw), *double-support time* (DS), their percentages (Sa%, Sw%, and DS%) of Sr, *step time* (St), *stride length* (SL), *gait speed* (GS), their values normalized to leg length (SL_n1_ and GS_n1_) and to body height (SL_n2_ and GS_n2_), and *cadence* (Cad).

		Women (n = 46)		Men (n = 55)		Men & Women (n = 101)		*p*
		Mean (SD)	95% CI	Mean (SD)	95% CI	Mean (SD)	95% CI	
FWP	Sr	−0.147 (0.067)	[−0.167, −0.127]	−0.161 (0.074)	[−0.182, −0.141]	−0.155 (0.071)	[−0.169, −0.141]	0.318
	Sa	−0.113 (0.049)	[−0.127, −0.098]	−0.125 (0.056)	[−0.140, −0.110]	−0.120 (0.053)	[−0.130, −0.109]	0.218
	Sw	−0.034 (0.020)	[−0.040, −0.028]	−0.036 (0.022)	[−0.042, −0.030]	−0.035 (0.021)	[−0.039, −0.031]	0.715
	DS	−0.040 (0.016)	[−0.045, −0.035]	−0.045 (0.020)	[−0.050, −0.039]	−0.042 (0.019)	[−0.046, −0.039]	0.134
	St	−0.074 (0.033)	[−0.084, −0.064]	−0.081 (0.037)	[−0.091, −0.071]	−0.077 (0.035)	[−0.084, −0.070]	0.338
	SL	0.211 (0.087)	[0.185, 0.237]	0.281 (0.121)	[0.248, 0.314]	0.249 (0.112)	[0.227, 0.271]	**0.001**
	GS	0.484 (0.178)	[0.431, 0.536]	0.597 (0.268)	[0.525, 0.670]	0.546 (0.237)	[0.499, 0.592]	**0.016**
	Cad	0.168 (0.085)	[0.142, 0.193]	0.176 (0.092)	[0.151, 0.201]	0.172 (0.088)	[0.155, 0.190]	0.631
	Sa%	−2.35 (0.94)	[−2.64, −2.07]	−2.77 (1.39)	[−3.14, −2.39]	−2.58 (1.22)	[−2.82, −2.34]	0.107
	Sw%	2.35 (0.94)	[2.07, 2.64]	2.77 (1.39)	[2.39, 3.14]	2.58 (1.22)	[2.34, 2.82]	0.107
	DS%	−2.36 (0.95)	[−2.64, −2.08]	−2.77 (1.38)	[−3.15, −2.40]	−2.58 (1.22)	[−2.82, −2.34]	0.084
	SL_n1_	0.245 (0.100)	[0.215, 0.274]	0.311 (0.139)	[0.274, 0.349]	0.281 (0.126)	[0.256, 0.306]	**0.006**
	GS_n1_	0.563 (0.214)	[0.500, 0.627]	0.661 (0.308)	[0.577, 0.744]	0.616 (0.272)	[0.563, 0.670]	0.074
	SL_n2_	0.129 (0.053)	[0.113, 0.145]	0.159 (0.067)	[0.141, 0.177]	0.145 (0.063)	[0.133, 0.158]	**0.017**
	GS_n2_	0.297 (0.110)	[0.264, 0.329]	0.337 (0.148)	[0.297, 0.377]	0.319 (0.133)	[0.293, 0.345]	0.125
FWP%	Sr	−14.3 (6.1)	[−16.1, −12.4]	−15.2 (6.5)	[−16.9, −13.4]	−14.8 (6.3)	[−16.0, −13.5]	0.472
	Sa	−17.4 (6.8)	[−19.4, −15.3]	−18.8 (7.4)	[−20.8, −16.8]	−18.1 (7.1)	[−19.5, −16.7]	0.286
	Sw	−8.9 (5.1)	[−10.4, −7.4]	−9.0 (5.4)	[−10.5, −7.6]	−9.0 (5.2)	[−10.0, −7.9]	0.932
	DS	−29.6 (9.5)	[−32.4, −26.7]	−33.4 (10.8)	[−36.4, −30.5]	−31.7 (10.4)	[−33.7, −29.6]	0.061
	St	−14.3 (6.1)	[−16.1, −12.5]	−15.1 (6.4)	[−16.9, −13.4]	−14.7 (6.3)	[−16.0, −13.5]	0.489
	SL	14.6 (6.5)	[12.7, 16.5]	18.1 (8.7)	[15.8, 20.5]	16.5 (7.9)	[15.0, 18.1]	**0.019**
	GS	34.6 (14.7)	[30.2, 39.0]	40.8 (21.7)	[34.9, 46.6]	38.0 (19.0)	[34.2, 41.7]	0.094
	Cad	17.3 (9.2)	[14.6, 20.0]	18.7 (10.3)	[15.9, 21.5]	18.1 (9.8)	[16.1, 20.0]	0.493
	Sa%	−3.7 (1.4)	[−4.2, −3.3]	−4.4 (2.1)	[−5.0, −3.8]	−4.1 (1.9)	[−4.5, −3.7]	0.069
	Sw%	6.4 (2.7)	[5.6, 7.2]	7.4 (4.1)	[6.3, 8.5]	7.0 (3.5)	[6.3, 7.7]	0.172
	DS%	−18.3 (6.3)	[−20.1, −16.4]	−22.1 (9.0)	[−24.5, −19.6]	−20.3 (8.1)	[−21.9, −18.7]	**0.017**

**Table 9:** Comparison between intra-sessions reliability results for some spatiotemporal gait parameters reported in healthy adults in previous studies and those obtained in the present study. The considered parameters are GS (*gait speed*) [m/s]; SL (*stride length*) [m]; SL_n1_ [dimensionless], where SL is normalized to leg length, and Sa% and Sw% are, respectively, Sa (*stance time*) and Sw (*swing time*) as percentages [%] of the stride time.

			ICC	SEM (SEM%)	MDC (MDC%)
Hars et al., 2013 [35]; n = 30 (30 men), age = 75.2 ± 6.9 years.	Preferred walking	GS	0.84	0.045 (4.30)	0.124 (11.80)
	Dual-task walking	GS	0.85	0.056 (5.90)	0.155 (16.30)
	Fast walking	GS	0.90	0.039 (2.7)	0.108 (7.5)
Bernal et al., 2016 [36]; n = 126 (85 men), age = 27.37 ± 1.77 years.	Preferred walking	SL	0.89	0.027 (-)	-
		GS	0.88	0.036 (-)	-
Soulard et al., 2021 [11]; n = 20 (10 men), age = 44.9 ± 11.7 years.	Preferred walking	SL	0.97	0.030 (1.74)	0.070 (4.82)
		GS	0.87	0.060 (3.79)	0.160 (10.50)
		SL_n1_	0.95	0.030 (1.70)	0.080 (4.72)
		Sa%	0.69	1.17 (1.95)	3.24 (5.40)
		Sw%	0.69	1.17 (2.93)	3.24 (8.11)
Present work; n = 101 (55 men), age = 52.0 ± 5.7 years.	Preferred walking	SL	0.99	0.014 (0.91)	0.039 (2.52)
		GS	0.98	0.023 (1.55)	0.063 (4.29)
		SL_n1_	0.99	0.015 (0.89)	0.042 (2.46)
		Sa%	0.99	0.20 (0.30)	0.50 (0.82)
		Sw%	0.99	0.20 (0.50)	0.50 (1.38)
	Dual-task walking	GS	0.97	0.041 (2.96)	0.113 (8.19)
	Fast walking	GS	0.95	0.056 (2.80)	0.156 (7.75)

Sign “-”: the parameter is not available or is not considered in the corresponding study.

## Data Availability

Data are contained within the article and Supplementary Materials.

## Supplementary Materials

The following supporting information can be downloaded at: https://www.mdpi.com/article/10.3390/s25041267/s1.
