# Supplementary material for: Toward Convenient and Accurate IMU-Based Gait Analysis
Source: Sensors (Basel). 2025 Feb 19;25(4):1267. doi: 10.3390/s25041267 (PMC11860383; doi:10.3390/s25041267)
Supplement: Supplementary file 1 [file sensors-25-01267-s001.zip › sensors-3434852-supplementary.pdf]

Toward Convenient and Accurate IMU-based Gait Analysis

Mohamed Boutaayamou[1,2,*], Doriane Pelzer[3], Cédric Schwartz[1], Sophie Gillain[4], Gaëtan Garraux[5], Jean-Louis Croisier[1], Jacques G. Verly[2], and Olivier Brüls[1]

[1]Laboratory of Movement Analysis, University of Liège, B-4000 Liège, Belgium,
[2]Department of Electrical Engineering and Computer Science, University of Liège, B-4000 Liège Belgium,
[3]Physical Medicine and Sport Traumatology Department, University Hospital of Liege, Liège, Belgium,
[4]Geriatrics Department, University Hospital of Liège, Liège, Belgium,
[5]Neurology Department, University Hospital of Liège, Liège, Belgium.

* Correspondence: mboutaayamou@uliege.com

Supplementary Material

TABLE S1. Comparison between the left and right values of the spatiotemporal gait parameters obtained in 101 healthy adults (55 men) during trials 1 and 2 at three walking speed conditions including preferred, dual-task, and fast walking. These parameters are *Stride time* (Sr) [s], *Stance time* (Sa) [s], *Swing time* (Sw) [s], *Double-Support time* (DS) [s], *Step time* (St) [s], *Stride length* (SL) [m], *Gait speed* (GS) [m/s], and *Cadence* (Cad) [strides/s]. Sa%, Sw%, and DS% are Sa, Sw, and DS as percentages [%] of Sr.

FIGURE S1. Bland-Altman plots and distributions of the *individual values* of the left and right spatiotemporal gait parameters obtained in trials 1 and 2 at preferred walking (PW). For a given parameter and side, the central dash-dotted line indicates the mean differences between *individual values* of this parameter from trials 1 and 2. The dashed line indicates the limits of agreement, which are ±1.96 standard deviations of these differences, showing the range within which 95% of the differences fall. The considered parameters are *Stride time* (Sr) [s], *Stance time* (Sa) [s], *Swing time* (Sw) [s], *Double-Support time* (DS) [s], *Step time* (St) [s], *Stride length* (SL) [m], *Gait speed* (GS) [m/s], and *Cadence* (Cad) [strides/s]. Sa%, Sw%, DS%, and St% are Sa, Sw, DS, and St as percentages [%] of Sr.

FIGURE S2. Bland-Altman plots and distributions of the *individual values* of the left and right spatiotemporal gait parameters obtained in trials 1 and 2 at dual-task walking (DTW). For a given parameter and side, the central dash-dotted line indicates the mean differences between *individual values* of this parameter from trials 1 and 2. The dashed line indicates the limits of agreement, which are ±1.96 standard deviations of these differences, showing the range within which 95% of the differences fall. The considered parameters are *Stride time* (Sr) [s], *Stance time* (Sa) [s], *Swing time* (Sw) [s], *Double-Support time* (DS) [s], *Step time* (St) [s], *Stride length* (SL) [m], *Gait speed* (GS) [m/s], and *Cadence* (Cad) [strides/s]. Sa%, Sw%, DS%, and St% are Sa, Sw, DS, and St as percentages [%] of Sr.

FIGURE S3. Bland-Altman plots and distributions of the *individual values* of the left and right spatiotemporal gait parameters obtained in trials 1 and 2 at fast walking (FW). For a given parameter and side, the central dash-dotted line indicates the mean differences between *individual values* of this parameter from trials 1 and 2. The dashed line indicates the limits of agreement, which are ±1.96 standard deviations of these differences, showing the range within which 95% of the differences fall. The considered parameters are *Stride time* (Sr) [s], *Stance time* (Sa) [s], *Swing time* (Sw) [s], *Double-Support time* (DS) [s], *Step time* (St) [s], *Stride length* (SL) [m], *Gait speed* (GS) [m/s], and *Cadence* (Cad) [strides/s]. Sa%, Sw%, DS%, and St% are Sa, Sw, DS, and St as percentages [%] of Sr.

FIGURE S4. Bland-Altman plots and distributions of the *average values* of the left and right spatiotemporal gait parameters in trials 1 and 2 at preferred walking (PW). For a given parameter and side, the central dash-dotted line indicates the mean differences between *average values* of this parameter from trials 1 and 2. The dashed line indicates the limits of agreement, which are ±1.96 standard deviations of these differences, showing the range within which 95% of the differences fall. The considered parameters are *Stride time* (Sr) [s], *Stance time* (Sa) [s], *Swing time* (Sw) [s], *Double-Support time* (DS) [s], *Step time* (St) [s], *Stride length* (SL) [m], *Gait speed* (GS) [m/s], and *Cadence* (Cad) [strides/s]. Sa%, Sw%, DS%, and St% are Sa, Sw, DS, and St as percentages [%] of Sr.

FIGURE S5. Bland-Altman plots and distributions of the *average values* of the left and right spatiotemporal gait parameters in trials 1 and 2 at dual-task walking (DTW). For a given parameter and side, the central dash-dotted line indicates the mean differences between *average values* of this parameter from trials 1 and 2. The dashed line indicates the limits of agreement, which are ±1.96 standard deviations of these differences, showing the range within which 95% of the differences fall. The considered parameters are *Stride time* (Sr) [s], *Stance time* (Sa) [s], *Swing time* (Sw) [s], *Double-Support time* (DS) [s], *Step time* (St) [s], *Stride length* (SL) [m], *Gait speed* (GS) [m/s], and *Cadence* (Cad) [strides/s]. Sa%, Sw%, DS%, and St% are Sa, Sw, DS, and St as percentages [%] of Sr.

FIGURE S6. Bland-Altman plots and distributions of the *average values* of the left and right spatiotemporal gait parameters in trials 1 and 2 at fast walking (FW). For a given parameter and side, the central dash-dotted line indicates the mean differences between *average values* of this parameter from trials 1 and 2. The dashed line indicates the limits of agreement, which are ±1.96 standard deviations of these differences, showing the range within which 95% of the differences fall. The considered parameters are *Stride time* (Sr) [s], *Stance time* (Sa) [s], *Swing time* (Sw) [s], *Double-Support time* (DS) [s], *Step time* (St) [s], *Stride length* (SL) [m], *Gait speed* (GS) [m/s], and *Cadence* (Cad) [strides/s]. Sa%, Sw%, DS%, and St% are Sa, Sw, DS, and St as percentages [%] of Sr.

TABLE S2. Intra-session reliability results for symmetry index SI1 in 101 healthy adults (55 men) at preferred, dual-task, and fast walking speeds. The index values were obtained by applying Formula 1 to *Stance time* (Sa), *Swing time* (Sw), *Double-Support time* (DS), *Step time* (St), their respective percentages (Sa%, Sw%, DS%, and St%) of Sr, *Stride length* (SL), and to *Gait speed* (GS).

TABLE S3. Intra-session reliability results for symmetry index SI2 in 101 healthy adults (55 men) at preferred, dual-task, and fast walking speeds. The index values were obtained by applying Formula 2 to *Stance time* (Sa), *Swing time* (Sw), *Double-Support time* (DS), *Step time* (St), their respective percentages (Sa%, Sw%, DS%, and St%) of Sr, *Stride length* (SL), and to *Gait speed* (GS).

TABLE S4. Intra-session reliability results for symmetry index SI3 in 101 healthy adults (55 men) at preferred, dual-task, and fast walking speeds. The index values were obtained by applying Formula 3 to *Stance time* (Sa), *Swing time* (Sw), *Double-Support time* (DS), *Step time* (St), their respective percentages (Sa%, Sw%, DS%, and St%) of Sr, *Stride length* (SL), and to *Gait speed* (GS).

TABLE S5. Intra-session reliability results of dual-task cost indices, DTC and DTC%, in 101 healthy adults (55 men). These indices are obtained for *Stride time* (Sr), *Stance time* (Sa), *Swing time* (Sw), *Double-Support time* (DS), *Step time* (St), their respective percentages (Sa%, Sw%, DS%, and St%) of Sr, *Stride length* (SL), *Gait speed* (GS), *Cadence* (Cad), SL and GS normalized to leg length (SL_{n1} and GS_{n1}) and to body height (SL_{n2} and GS_{n2}).

TABLE S6. Reference values for spatiotemporal gait parameters and symmetry ratio SI4, and the results of the following parameter comparisons: (1) preferred walking (PW) vs dual-task walking (DTW), (2) PW vs fast walking (FW), and (3) DTW vs FW. These parameters are *Stride time* (Sr) [s], *Stance time* (Sa) [s], *Swing time* (Sw) [s], *Double-Support time* (DS) [s], *Step time* (St) [s], their respective percentages (Sa%, Sw%, DS%, and St%) of Sr, *Stride length* (SL) [m], *Gait speed* (GS) [m/s], their respective normalized values to leg length (SL_{n1} [dimensionless] and GS_{n1} [s^{-1}]) and to body height (SL_{n2} [dimensionless] and GS_{n2} [s^{-1}]), and *Cadence* (Cad) [strides/s].

TABLE S1. Comparison between the left and right values of the spatiotemporal gait parameters obtained in 101 healthy adults (55 men) during trials 1 and 2 at three walking speed conditions including preferred, dual-task, and fast walking. These parameters are *Stride time* (Sr) [s], *Stance time* (Sa) [s], *Swing time* (Sw) [s], *Double-Support time* (DS) [s], *Step time* (St) [s], *Stride length* (SL) [m], *Gait speed* (GS) [m/s], and *Cadence* (Cad) [strides/s]. Sa%, Sw%, and DS% are Sa, Sw, and DS as percentages [%] of Sr.

		Trial 1			Trial 2		
		Left side Mean (SD)	Right side Mean (SD)	*p-value*	Left side Mean (SD)	Right side Mean (SD)	*p-value*
**Preferred walking**	Sr	1.040 (0.054)	1.040 (0.054)	0.99	1.043 (0.054)	1.043 (0.053)	0.93
	Sa	0.650 (0.042)	0.651 (0.043)	0.78	0.653 (0.041)	0.653 (0.042)	0.97
	Sw	0.390 (0.021)	0.389 (0.022)	0.79	0.390 (0.022)	0.390 (0.021)	1.00
	DS	0.129 (0.020)	0.132 (0.021)	0.24	0.130 (0.019)	0.133 (0.020)	0.21
	St	0.519 (0.028)	0.521 (0.027)	0.57	0.520 (0.028)	0.523 (0.027)	0.26
	SL	1.534 (0.140)	1.524 (0.147)	0.64	1.531 (0.138)	1.520 (0.142)	0.58
	GS	1.479 (0.151)	1.470 (0.158)	0.65	1.473 (0.144)	1.462 (0.150)	0.60
	Cad	0.965 (0.049)	0.965 (0.049)	0.99	0.962 (0.048)	0.962 (0.048)	0.98
	Sa%	62.5 (1.6)	62.6 (1.6)	0.73	62.6 (1.5)	62.6 (1.6)	0.98
	Sw%	37.5 (1.6)	37.4 (1.6)	0.73	37.4 (1.5)	37.4 (1.6)	0.98
	DS%	12.3 (1.5)	12.7 (1.6)	0.14	12.4 (1.5)	12.7 (1.6)	0.11
**Dual-task walking**	Sr	1.091 (0.135)	1.090 (0.132)	0.99	1.088 (0.116)	1.089 (0.116)	0.98
	Sa	0.685 (0.089)	0.687 (0.091)	0.89	0.685 (0.078)	0.685 (0.078)	0.97
	Sw	0.406 (0.050)	0.404 (0.046)	0.91	0.403 (0.042)	0.402 (0.040)	0.98
	DS	0.139 (0.027)	0.142 (0.029)	0.52	0.139 (0.026)	0.143 (0.026)	0.30
	St	0.545 (0.068)	0.546 (0.066)	0.89	0.542 (0.058)	0.546 (0.058)	0.48
	SL	1.495 (0.159)	1.485 (0.165)	0.65	1.488 (0.157)	1.478 (0.164)	0.68
	GS	1.391 (0.232)	1.381 (0.232)	0.75	1.385 (0.218)	1.376 (0.222)	0.75
	Cad	0.928 (0.098)	0.929 (0.097)	0.99	0.929 (0.089)	0.929 (0.089)	0.92
	Sa%	62.8 (1.7)	62.9 (1.6)	0.55	62.9 (1.7)	62.9 (1.7)	0.99
	Sw%	37.2 (1.7)	37.1 (1.6)	0.55	37.1 (1.7)	37.1 (1.7)	0.99
	DS%	12.7 (1.6)	12.9 (1.7)	0.31	12.7 (1.6)	13.1 (1.7)	0.11
**Fast walking**	Sr	0.882 (0.065)	0.882 (0.065)	0.98	0.890 (0.065)	0.890 (0.065)	0.99
	Sa	0.528 (0.043)	0.529 (0.044)	0.97	0.535 (0.043)	0.535 (0.045)	0.95
	Sw	0.354 (0.026)	0.354 (0.026)	0.90	0.355 (0.026)	0.355 (0.026)	0.92
	DS	0.086 (0.015)	0.089 (0.016)	0.10	0.089 (0.015)	0.092 (0.016)	0.13
	St	0.440 (0.033)	0.443 (0.032)	0.50	0.443 (0.034)	0.447 (0.032)	0.42
	SL	1.794 (0.172)	1.784 (0.179)	0.69	1.769 (0.164)	1.761 (0.173)	0.73
	GS	2.045 (0.252)	2.033 (0.257)	0.63	1.998 (0.228)	1.991 (0.247)	0.60
	Cad	1.141 (0.089)	1.140 (0.088)	0.93	1.131 (0.086)	1.130 (0.086)	0.98
	Sa%	59.9 (1.4)	59.9 (1.5)	0.95	60.1 (1.3)	60.1 (1.5)	0.75
	Sw%	40.1 (1.4)	40.1 (1.5)	0.95	39.9 (1.3)	39.9 (1.5)	0.75
	DS%	9.6 (1.4)	10.0 (1.5)	0.06	9.9 (1.4)	10.3 (1.5)	0.06

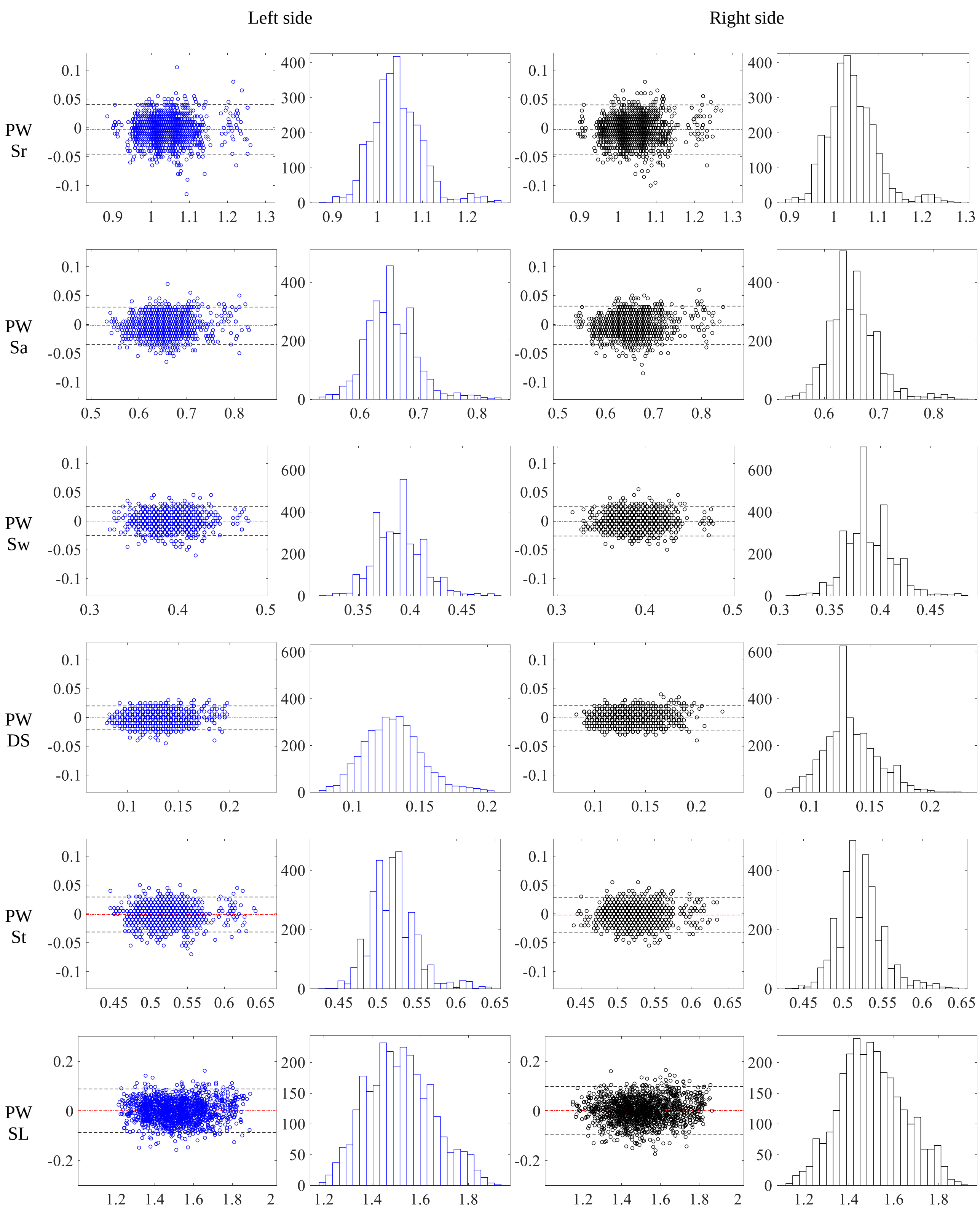
Left side
Right side
PW Sr
PW Sa
PW Sw
PW DS
PW St
PW SL

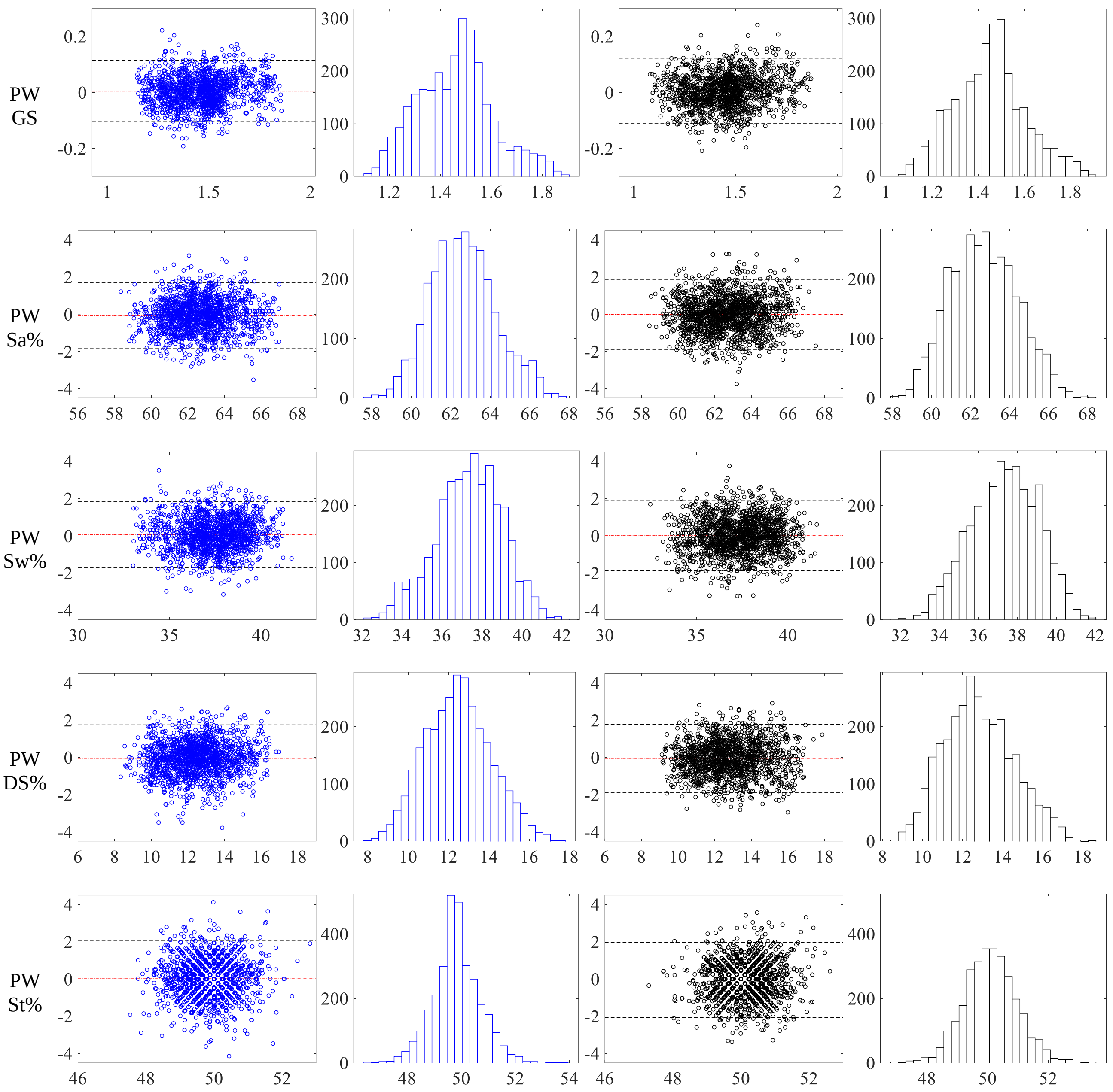

FIGURE 1. Bland-Altman plots and distributions of the *individual values* of the left and right spatiotemporal gait parameters obtained in trials 1 and 2 at preferred walking (PW). For a given parameter and side, the central dash-dotted line indicates the mean differences between *individual values* of this parameter from trials 1 and 2. The dashed line indicates the limits of agreement, which are ±1.96 standard deviations of these differences, showing the range within which 95% of the differences fall. The considered parameters are *Stride time* (Sr) [s], *Stance time* (Sa) [s], *Swing time* (Sw) [s], *Double-Support time* (DS) [s], *Step time* (St) [s], *Stride length* (SL) [m], *Gait speed* (GS) [m/s], and *Cadence* (Cad) [strides/s]. Sa%, Sw%, DS%, and St% are Sa, Sw, DS, and St as percentages [%] of Sr.

Toward Convenient and Accurate IMU-based Gait Analysis: Supplementary Material

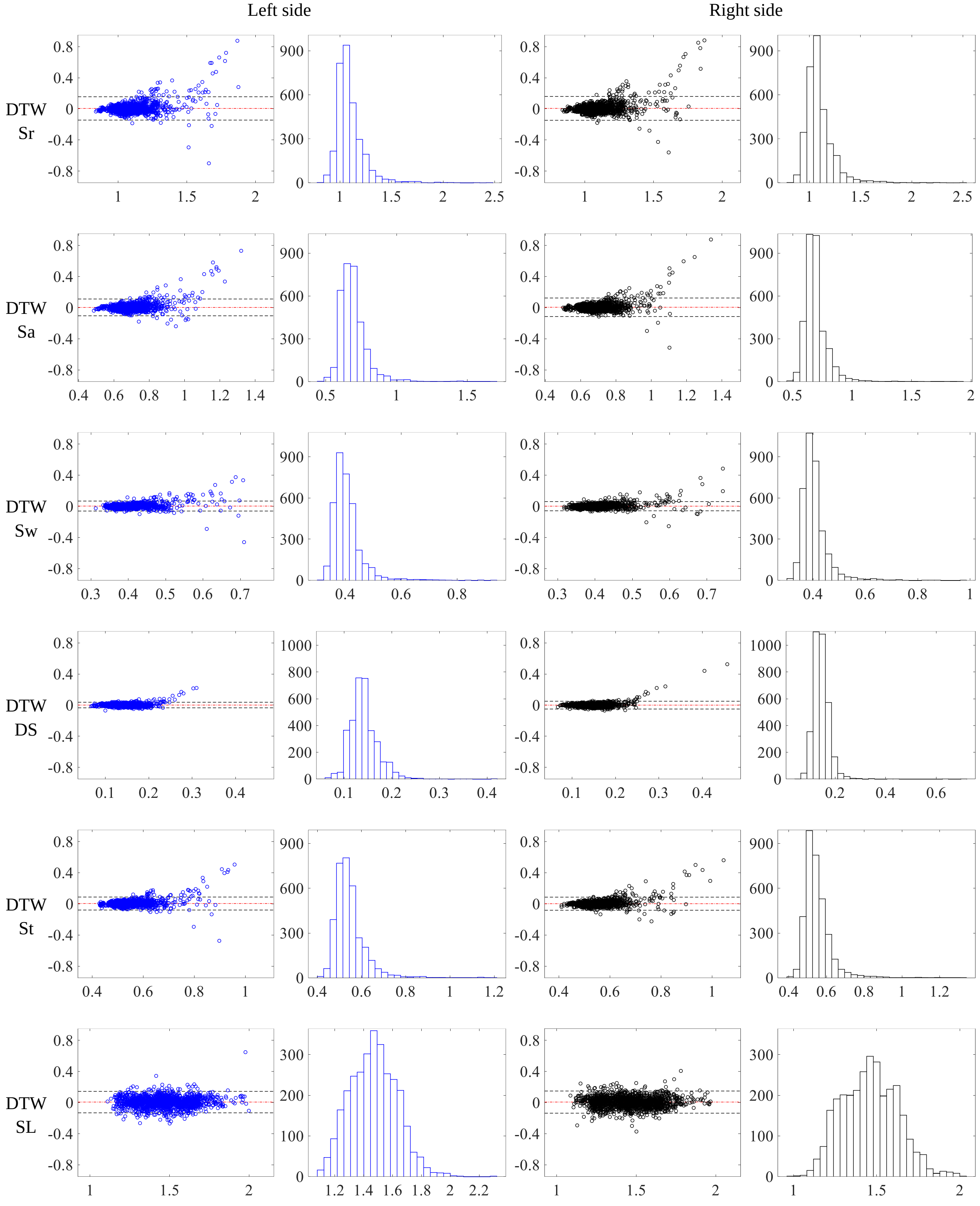

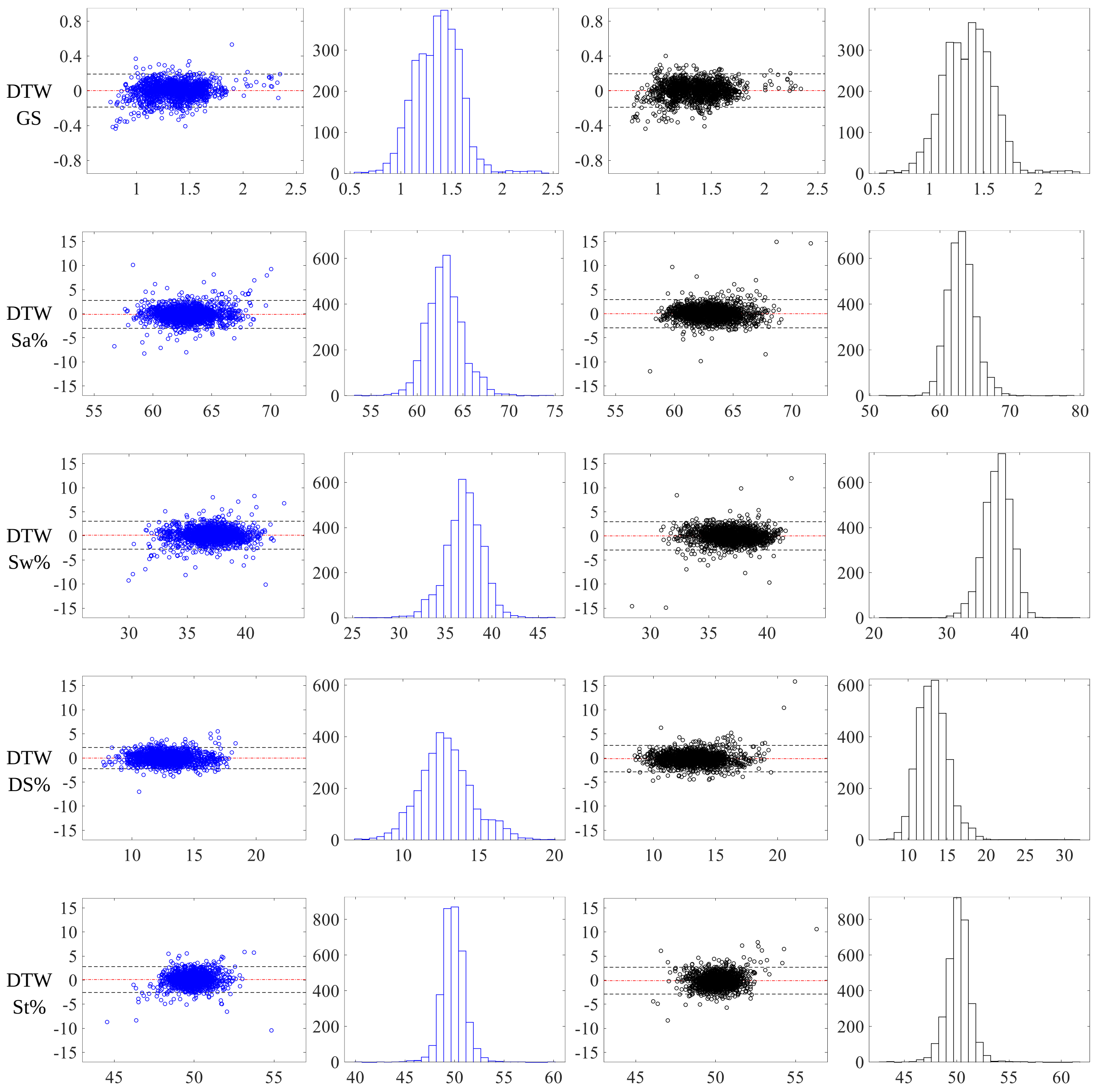

FIGURE S2. Bland-Altman plots and distributions of the *individual values* of the left and right spatiotemporal gait parameters obtained in trials 1 and 2 at dual-task walking (DTW). For a given parameter and side, the central dash-dotted line indicates the mean differences between *individual values* of this parameter from trials 1 and 2. The dashed line indicates the limits of agreement, which are ±1.96 standard deviations of these differences, showing the range within which 95% of the differences fall. The considered parameters are *Stride time* (Sr) [s], *Stance time* (Sa) [s], *Swing time* (Sw) [s], *Double-Support time* (DS) [s], *Step time* (St) [s], *Stride length* (SL) [m], *Gait speed* (GS) [m/s], and *Cadence* (Cad) [strides/s]. Sa%, Sw%, DS%, and St% are Sa, Sw, DS, and St as percentages [%] of Sr.

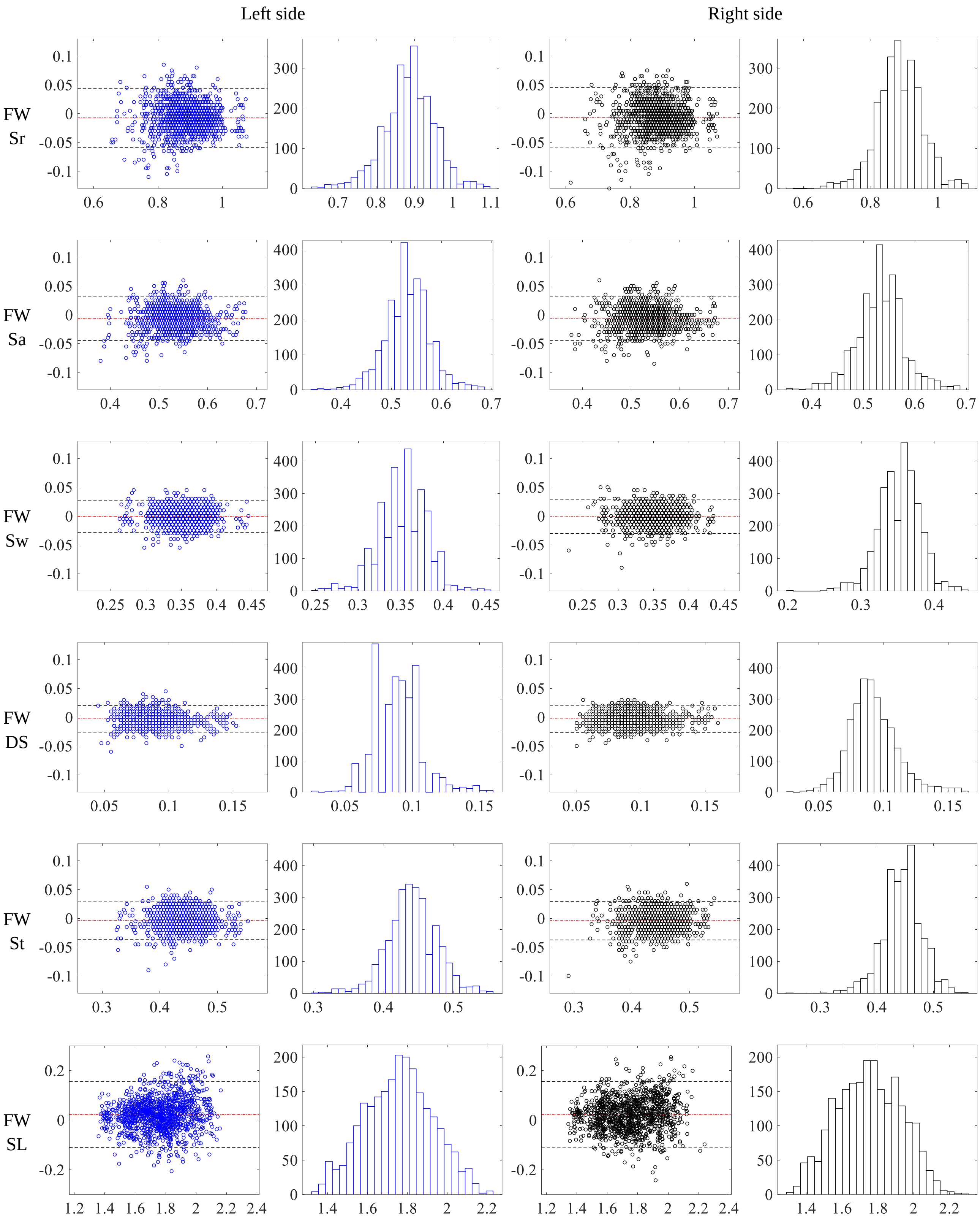
Left side
Right side
FW
Sr
FW
Sa
FW
Sw
FW
DS
FW
St
FW
SL

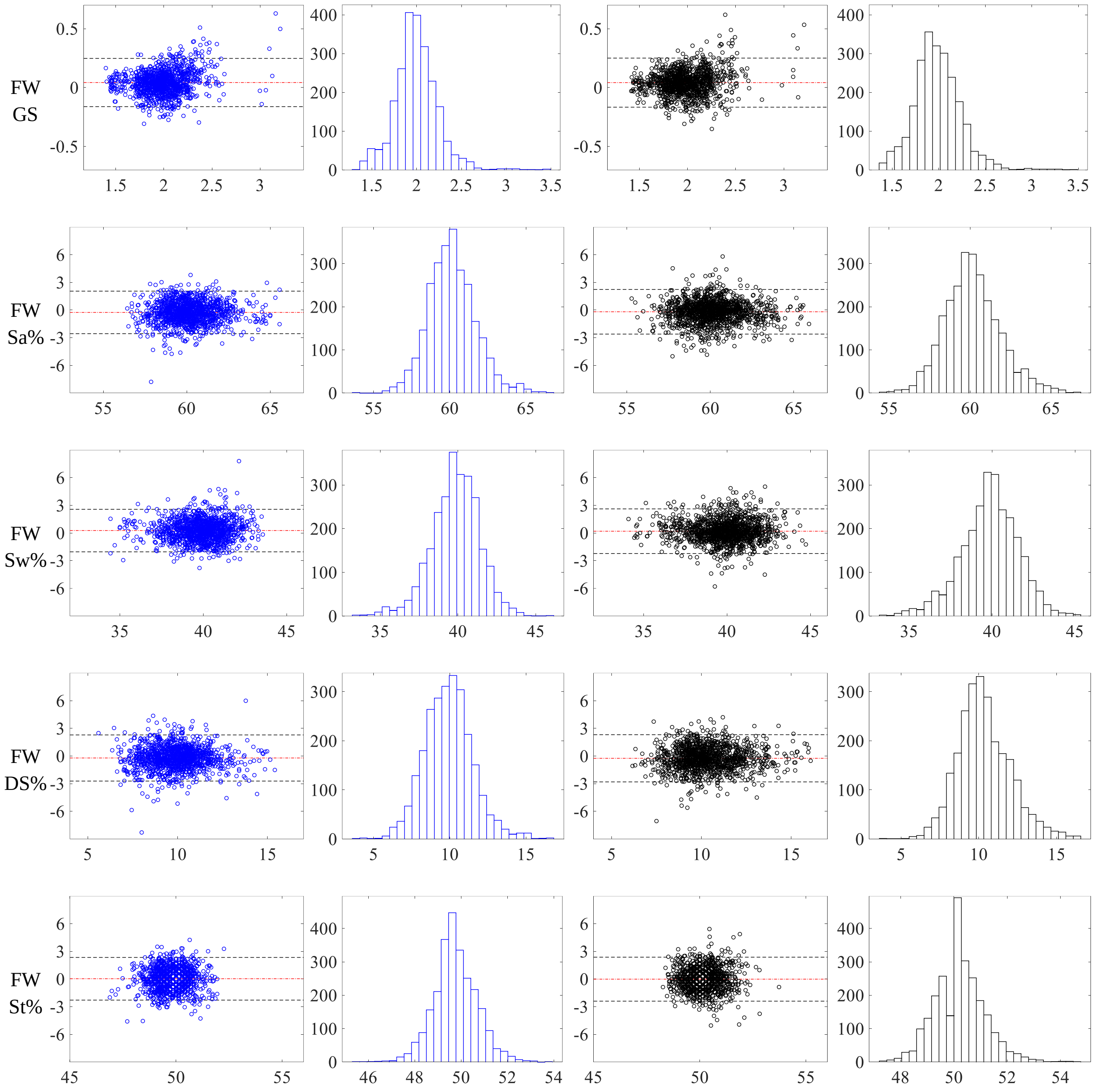

FIGURE S3. Bland-Altman plots and distributions of the *individual values* of the left and right spatiotemporal gait parameters obtained in trials 1 and 2 at fast walking (FW). For a given parameter and side, the central dash-dotted line indicates the mean differences between *individual values* of this parameter from trials 1 and 2. The dashed line indicates the limits of agreement, which are ±1.96 standard deviations of these differences, showing the range within which 95% of the differences fall. The considered parameters are *Stride time* (Sr) [s], *Stance time* (Sa) [s], *Swing time* (Sw) [s], *Double-Support time* (DS) [s], *Step time* (St) [s], *Stride length* (SL) [m], *Gait speed* (GS) [m/s], and *Cadence* (Cad) [strides/s]. Sa%, Sw%, DS%, and St% are Sa, Sw, DS, and St as percentages [%] of Sr.

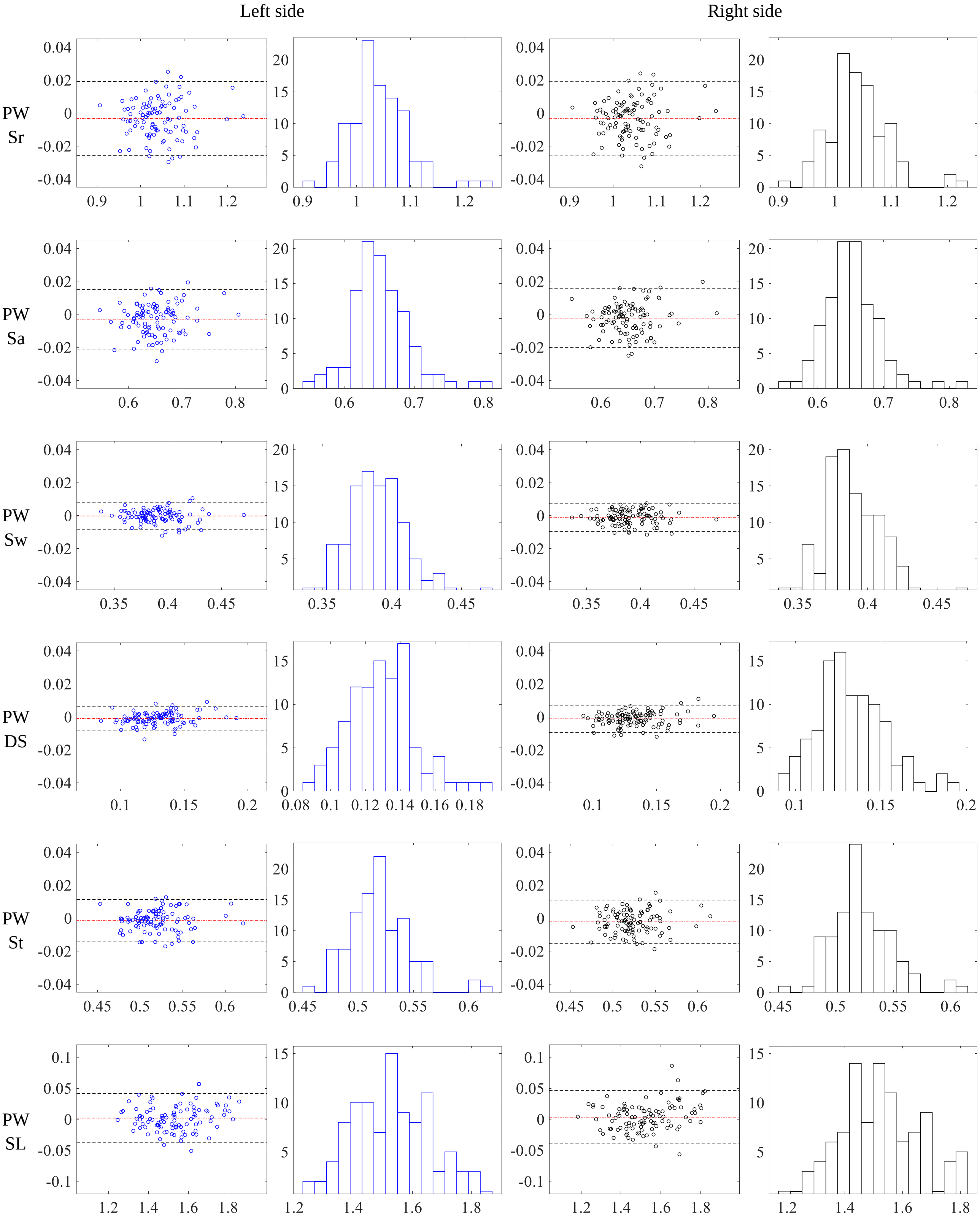
Left side
Right side
PW Sr
PW Sa
PW Sw
PW DS
PW St
PW SL

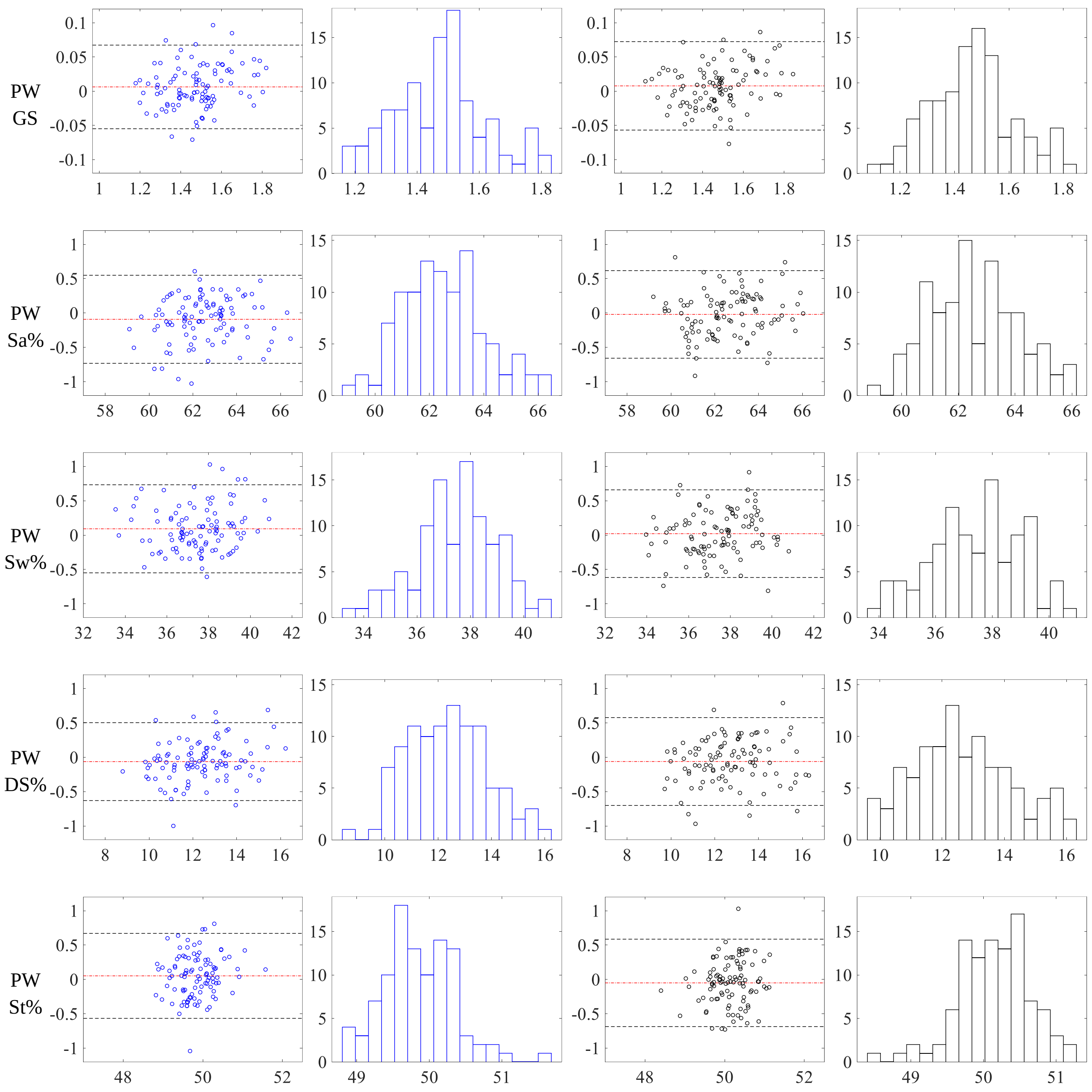

FIGURE S4. Bland-Altman plots and distributions of the *average values* of the left and right spatiotemporal gait parameters in trials 1 and 2 at preferred walking (PW). For a given parameter and side, the central dash-dotted line indicates the mean differences between *average values* of this parameter from trials 1 and 2. The dashed line indicates the limits of agreement, which are ±1.96 standard deviations of these differences, showing the range within which 95% of the differences fall. The considered parameters are *Stride time* (Sr) [s], *Stance time* (Sa) [s], *Swing time* (Sw) [s], *Double-Support time* (DS) [s], *Step time* (St) [s], *Stride length* (SL) [m], *Gait speed* (GS) [m/s], and *Cadence* (Cad) [strides/s]. Sa%, Sw%, DS%, and St% are Sa, Sw, DS, and St as percentages [%] of Sr.

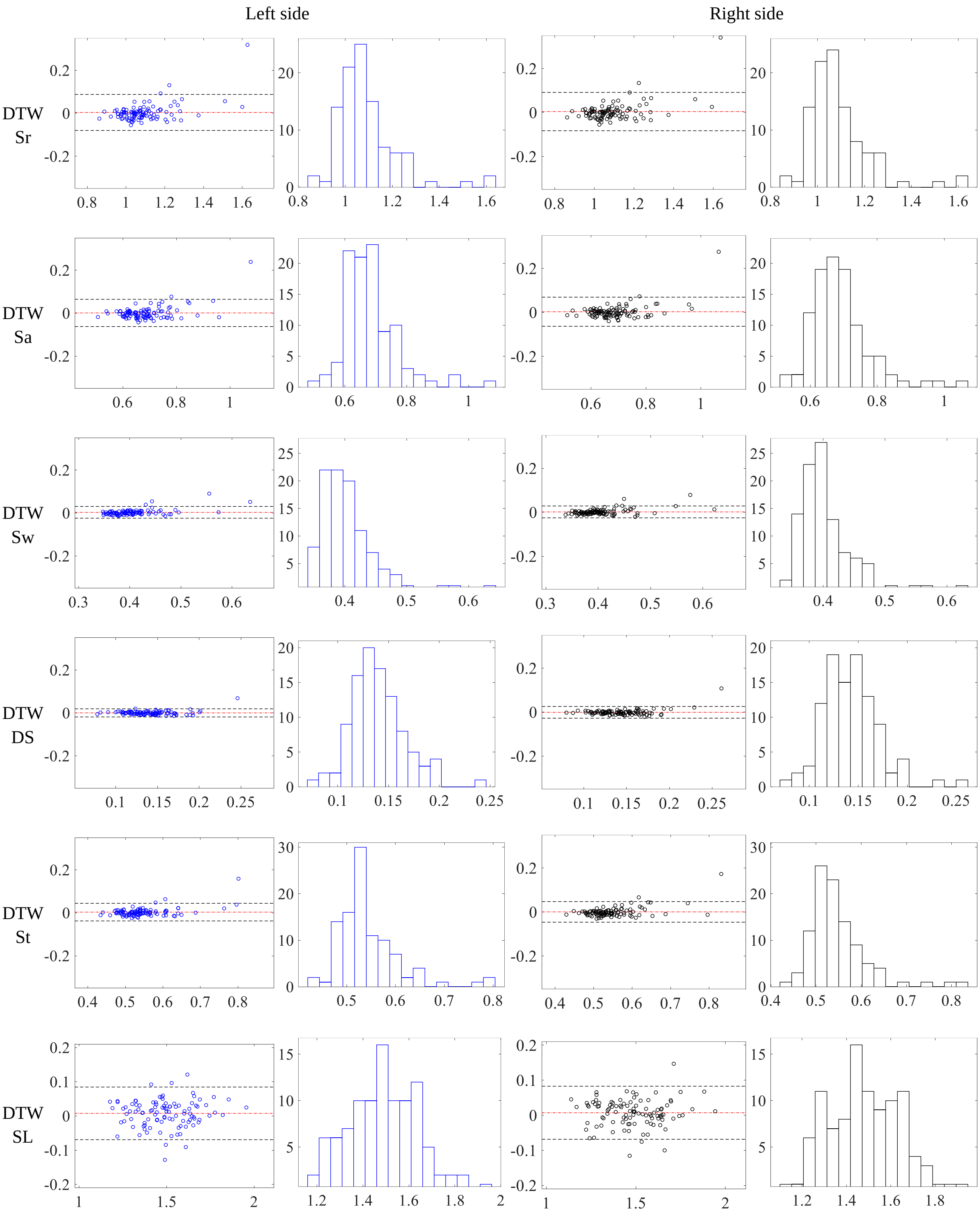

Left side
Right side
DTW Sr
DTW Sa
DTW Sw
DTW DS
DTW St
DTW SL

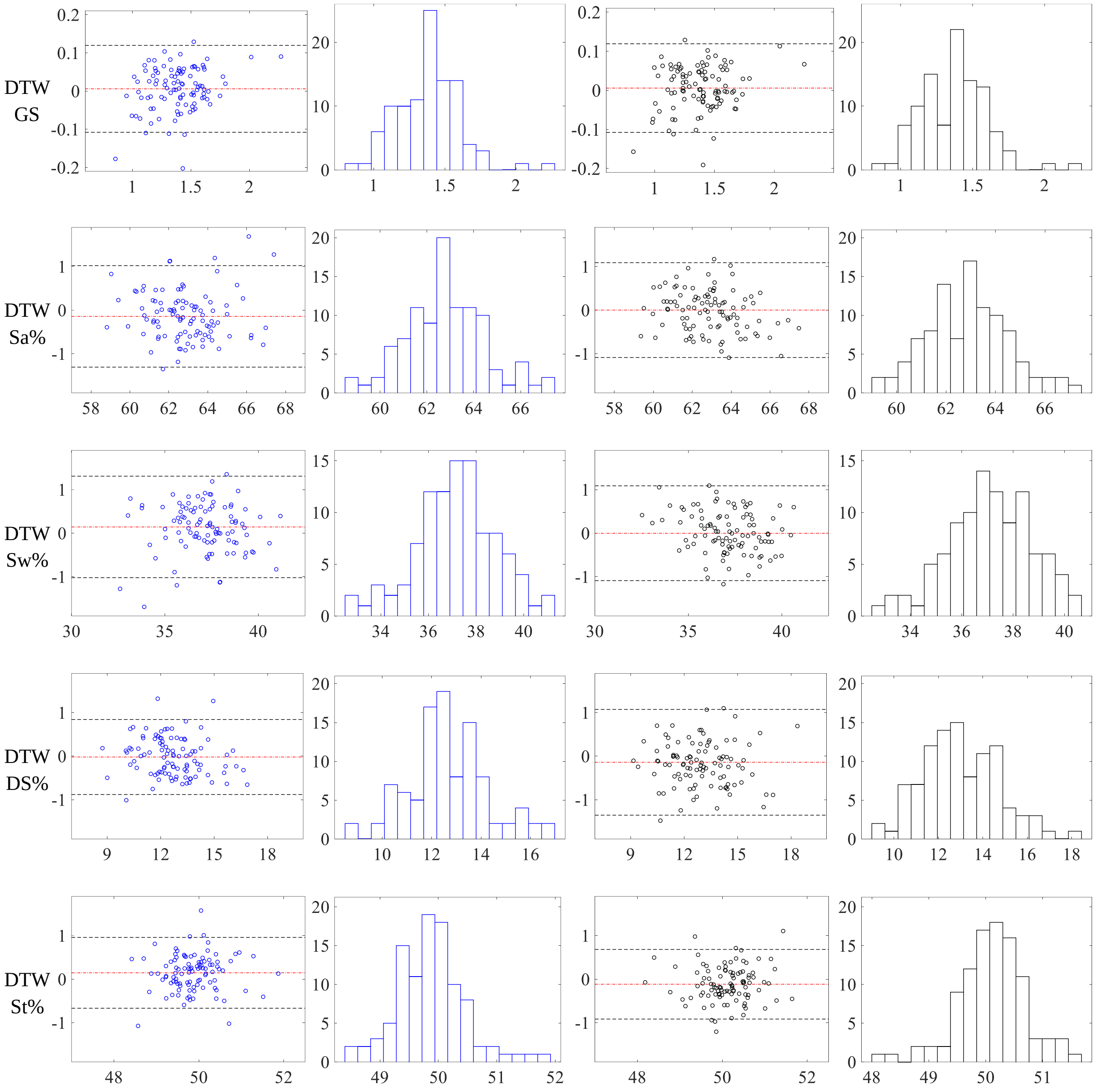

FIGURE S5. Bland-Altman plots and distributions of the *average values* of the left and right spatiotemporal gait parameters in trials 1 and 2 at dual-task walking (DTW). For a given parameter and side, the central dash-dotted line indicates the mean differences between *average values* of this parameter from trials 1 and 2. The dashed line indicates the limits of agreement, which are ±1.96 standard deviations of these differences, showing the range within which 95% of the differences fall. The considered parameters are *Stride time* (Sr) [s], *Stance time* (Sa) [s], *Swing time* (Sw) [s], *Double-Support time* (DS) [s], *Step time* (St) [s], *Stride length* (SL) [m], *Gait speed* (GS) [m/s], and *Cadence* (Cad) [strides/s]. Sa%, Sw%, DS%, and St% are Sa, Sw, DS, and St as percentages [%] of Sr.

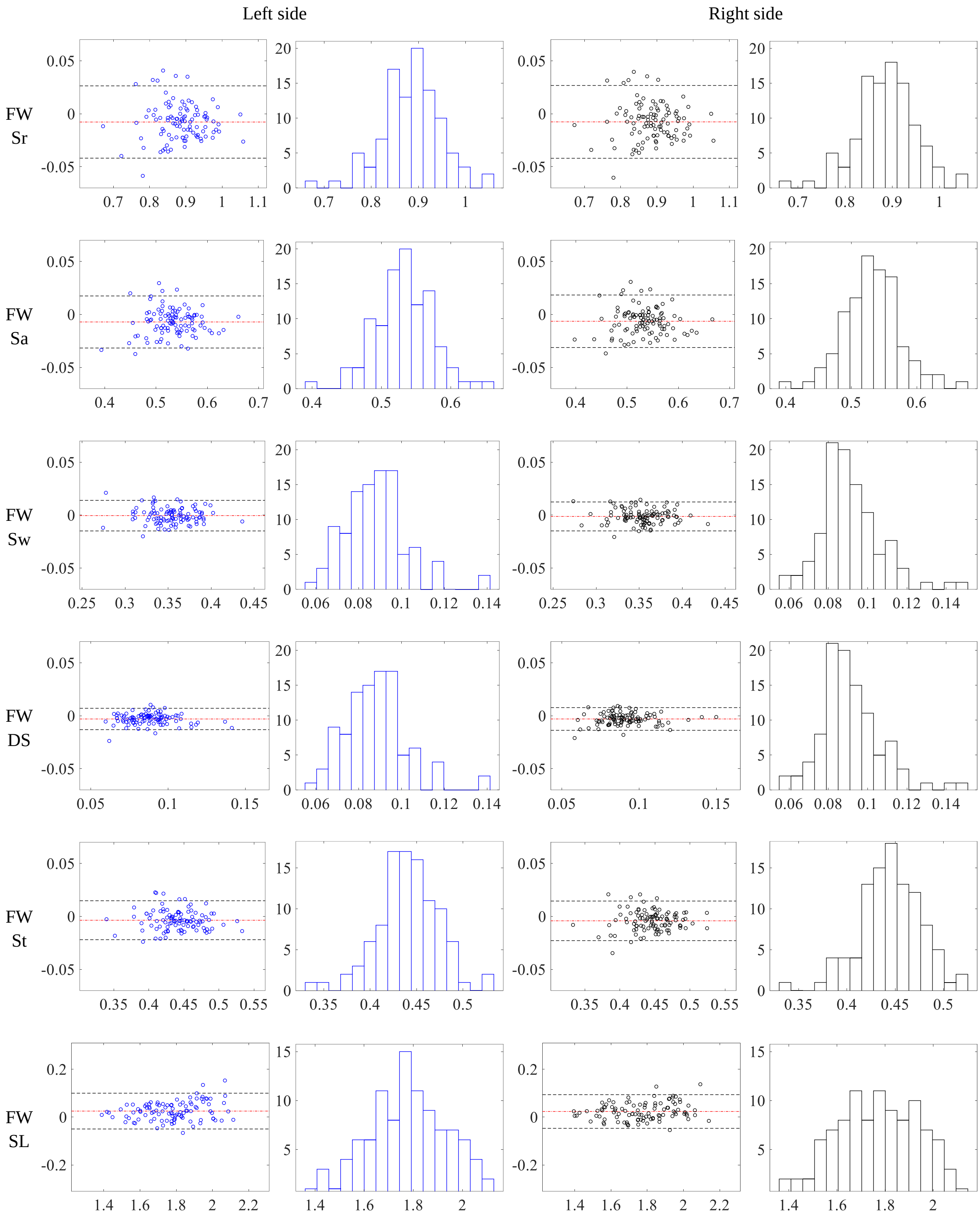
Left side
Right side
FW Sr
FW Sa
FW Sw
FW DS
FW St
FW SL

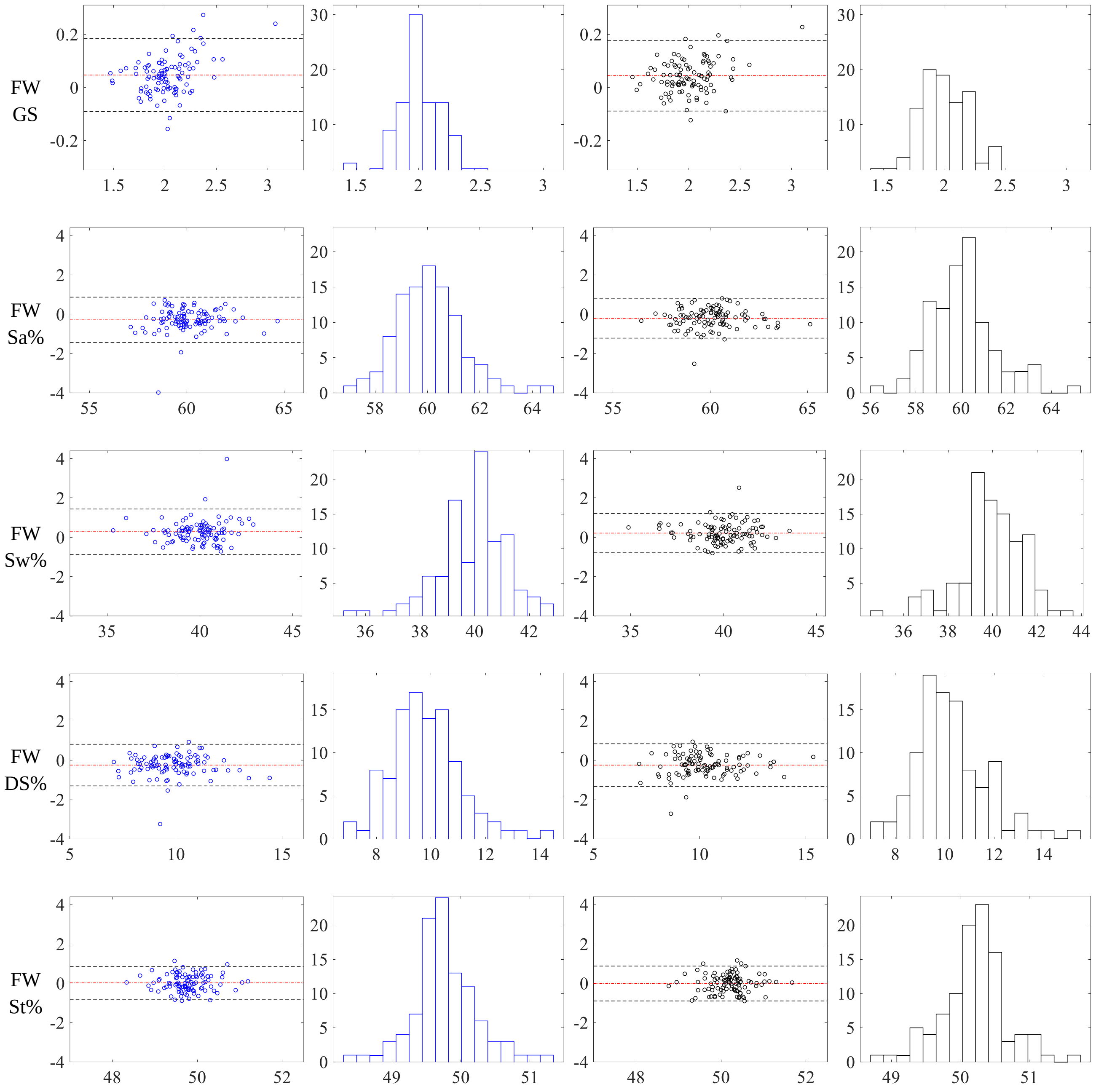

FIGURE S6. Bland-Altman plots and distributions of the *average values* of the left and right spatiotemporal gait parameters in trials 1 and 2 at fast walking (FW). For a given parameter and side, the central dash-dotted line indicates the mean differences between *average values* of this parameter from trials 1 and 2. The dashed line indicates the limits of agreement, which are ±1.96 standard deviations of these differences, showing the range within which 95% of the differences fall. The considered parameters are *Stride time* (Sr) [s], *Stance time* (Sa) [s], *Swing time* (Sw) [s], *Double-Support time* (DS) [s], *Step time* (St) [s], *Stride length* (SL) [m], *Gait speed* (GS) [m/s], and *Cadence* (Cad) [strides/s]. Sa%, Sw%, DS%, and St% are Sa, Sw, DS, and St as percentages [%] of Sr.

TABLE S2. Intra-session reliability results for symmetry index SI1 in 101 healthy adults (55 men) at preferred, dual-task, and fast walking speeds. The index values were obtained by applying Formula 1 to *Stance time* (Sa), *Swing time* (Sw), *Double-Support time* (DS), *Step time* (St), their respective percentages (Sa%, Sw%, DS%, and St%) of Sr, *Stride length* (SL), and to *Gait speed* (GS).

	Trial1: mean (SD)	Trial2: mean (SD)	ICC(2.1) [95% CI]	LOA [95% CI]	SEM (SEM%)	MDC (MDC%)	*p*
**Intra-session reliability results of SI1 at preferred walking**							
Sa	1.48 (0.57)	1.43 (0.61)	0.52 [0.36, 0.65]	[-1.19, 1.10]	0.41 (28.3)	1.14 (78.5)	0.38
Sw	2.44 (0.99)	2.38 (0.96)	0.58 [0.43, 0.69]	[-1.82, 1.71]	0.63 (26.3)	1.76 (73.0)	0.69
DS	7.15 (3.31)	6.79 (3.22)	0.72 [0.61, 0.80]	[-5.16, 4.45]	1.74 (24.9)	4.82 (69.1)	0.56
St	2.46 (1.08)	2.56 (1.09)	0.53 [0.38, 0.66]	[-1.96, 2.16]	0.74 (29.5)	2.06 (81.9)	0.36
SL	2.86 (1.80)	2.98 (1.85)	0.85 [0.79, 0.90]	[-1.81, 2.07]	0.70 (24.0)	1.94 (66.4)	0.43
GS	3.03 (1.79)	3.07 (1.77)	0.84 [0.77, 0.89]	[-1.95, 2.02]	0.71 (23.3)	1.97 (64.7)	0.73
Sa%	1.36 (0.56)	1.32 (0.55)	0.64 [0.51, 0.74]	[-0.96, 0.88]	0.33 (24.7)	0.92 (68.5)	0.52
Sw%	2.27 (0.98)	2.22 (0.95)	0.67 [0.54, 0.76]	[-1.59, 1.49]	0.55 (24.7)	1.53 (68.4)	0.70
DS%	7.22 (3.23)	6.79 (3.22)	0.74 [0.64, 0.82]	[-4.95, 4.09]	1.64 (23.5)	4.56 (65.1)	0.34
St%	2.31 (1.12)	2.43 (0.97)	0.53 [0.38, 0.66]	[-1.87, 2.10]	0.72 (30.2)	1.99 (83.8)	0.19
**Intra-session reliability results of SI1 at dual-task walking**							
Sa	2.03 (1.20)	1.81 (1.03)	0.69 [0.56, 0.78]	[-1.92, 1.49]	0.63 (32.7)	1.74 (90.6)	0.11
Sw	3.09 (1.53)	2.88 (1.56)	0.57 [0.42, 0.69]	[-3.01, 2.59]	1.01 (34.0)	2.81 (94.3)	0.21
DS	7.25 (3.19)	7.37 (3.24)	0.67 [0.55, 0.77]	[-5.00, 5.23]	1.84 (25.1)	5.09 (69.6)	0.89
St	3.10 (1.53)	3.09 (1.55)	0.66 [0.54, 0.76]	[-2.49, 2.46]	0.89 (28.7)	2.46 (79.6)	0.76
SL	3.57 (2.28)	3.53 (2.33)	0.89 [0.84, 0.92]	[-2.17, 2.09]	0.76 (21.5)	2.11 (59.5)	0.79
GS	3.83 (2.27)	3.73 (2.34)	0.86 [0.80, 0.90]	[-2.47, 2.28]	0.85 (22.6)	2.37 (62.6)	0.62
Sa%	1.72 (0.86)	1.56 (0.85)	0.62 [0.49, 0.73]	[-1.59, 1.27]	0.52 (31.8)	1.45 (88.2)	0.12
Sw%	2.94 (1.51)	2.66 (1.45)	0.62 [0.48, 0.72]	[-2.79, 2.24]	0.92 (32.8)	2.55 (91.0)	0.13
DS%	7.27 (3.27)	7.36 (3.26)	0.63 [0.49, 0.73]	[-5.45, 5.63]	1.99 (27.2)	5.51 (75.3)	0.98
St%	2.84 (1.24)	2.78 (1.44)	0.68 [0.56, 0.78]	[-2.16, 2.03]	0.75 (26.8)	2.09 (74.2)	0.36
**Intra-session reliability results of SI1 at fast walking**							
Sa	1.86 (0.77)	1.83 (0.85)	0.56 [0.41, 0.68]	[-1.52, 1.46]	0.54 (29.1)	1.49 (80.6)	0.61
Sw	2.79 (1.12)	2.76 (1.33)	0.50 [0.33, 0.63]	[-2.45, 2.40]	0.87 (31.4)	2.41 (87.0)	0.55
DS	10.62 (5.52)	9.99 (4.66)	0.56 [0.41, 0.68]	[-9.99, 8.73]	3.38 (32.8)	9.38 (91.0)	0.74
St	2.64 (1.23)	2.66 (1.19)	0.41 [0.23, 0.56]	[-2.56, 2.60]	0.93 (35.0)	2.57 (96.9)	0.89
SL	3.03 (1.58)	2.96 (1.53)	0.74 [0.64, 0.82]	[-2.26, 2.12]	0.79 (26.3)	2.18 (72.9)	0.71
GS	3.18 (1.55)	3.09 (1.58)	0.74 [0.64, 0.82]	[-2.31, 2.13]	0.80 (25.5)	2.21 (70.6)	0.45
Sa%	1.69 (0.73)	1.65 (0.78)	0.61 [0.47, 0.72]	[-1.35, 1.28]	0.47 (28.4)	1.31 (78.6)	0.49
Sw%	2.52 (1.10)	2.49 (1.22)	0.61 [0.47, 0.72]	[-2.04, 1.98]	0.72 (28.7)	2.00 (79.6)	0.54
DS%	10.72 (5.55)	9.89 (4.62)	0.56 [0.41, 0.68]	[-10.17, 8.50]	3.39 (32.9)	9.40 (91.2)	0.56
St%	2.51 (1.12)	2.47 (1.17)	0.41 [0.24, 0.56]	[-2.47, 2.39]	0.87 (35.1)	2.42 (97.2)	0.52

TABLE S3. Intra-session reliability results for symmetry index SI2 in 101 healthy adults (55 men) at preferred, dual-task, and fast walking speeds. The index values were obtained by applying Formula 2 to *Stance time* (Sa), *Swing time* (Sw), *Double-Support time* (DS), *Step time* (St), their respective percentages (Sa%, Sw%, DS%, and St%) of Sr, *Stride length* (SL), and to *Gait speed* (GS).

	Trial1: mean (SD)	Trial2: mean (SD)	ICC(2.1) [95% CI]	LOA [95% CI]	SEM (SEM%)	MDC (MDC%)	*p*
**Intra-session reliability results of SI2 at preferred walking**							
Sa	0.985 (0.006)	0.986 (0.006)	0.491 [0.328, 0.626]	[-0.011, 0.012]	0.004 (0.4)	0.011 (1.2)	0.34
Sw	0.976 (0.010)	0.977 (0.009)	0.576 [0.429, 0.693]	[-0.017, 0.018]	0.006 (0.6)	0.017 (1.7)	0.70
DS	0.932 (0.030)	0.934 (0.030)	0.724 [0.616, 0.805]	[-0.042, 0.045]	0.016 (1.7)	0.043 (4.6)	0.85
St	0.976 (0.010)	0.975 (0.011)	0.496 [0.334, 0.630]	[-0.021, 0.020]	0.007 (0.8)	0.021 (2.1)	0.44
SL	0.972 (0.017)	0.971 (0.018)	0.847 [0.781, 0.894]	[-0.020, 0.018]	0.007 (0.7)	0.019 (1.9)	0.44
GS	0.970 (0.017)	0.970 (0.017)	0.839 [0.770, 0.888]	[-0.019, 0.019]	0.007 (0.7)	0.019 (1.9)	0.75
Sa%	0.987 (0.005)	0.987 (0.005)	0.63 [0.50, 0.74]	[-0.009, 0.009]	0.003 (0.3)	0.009 (0.9)	0.55
Sw%	0.978 (0.009)	0.978 (0.009)	0.66 [0.53, 0.76]	[-0.014, 0.016]	0.005 (0.5)	0.015 (1.5)	0.66
DS%	0.931 (0.029)	0.935 (0.029)	0.75 [0.65, 0.83]	[-0.036, 0.043]	0.014 (1.5)	0.040 (4.3)	0.41
St%	0.977 (0.011)	0.976 (0.009)	0.53 [0.37, 0.66]	[-0.020, 0.018]	0.007 (0.7)	0.019 (2.0)	0.19
**Intra-session reliability results of SI2 at dual-task walking**							
Sa	0.980 (0.012)	0.982 (0.010)	0.68 [0.56, 0.78]	[-0.014, 0.019]	0.006 (0.6)	0.017 (1.7)	0.09
Sw	0.970 (0.015)	0.972 (0.015)	0.58 [0.44, 0.70]	[-0.024, 0.028]	0.010 (1.0)	0.026 (2.7)	0.17
DS	0.931 (0.029)	0.930 (0.029)	0.65 [0.52, 0.75]	[-0.049, 0.046]	0.017 (1.8)	0.047 (5.1)	0.78
St	0.969 (0.015)	0.970 (0.015)	0.66 [0.54, 0.76]	[-0.023, 0.024]	0.009 (0.9)	0.024 (2.4)	0.80
SL	0.965 (0.021)	0.965 (0.022)	0.88 [0.83, 0.92]	[-0.021, 0.021]	0.007 (0.8)	0.021 (2.1)	0.84
GS	0.963 (0.021)	0.964 (0.022)	0.86 [0.79, 0.90]	[-0.022, 0.023]	0.008 (0.8)	0.023 (2.3)	0.70
Sa%	0.983 (0.008)	0.985 (0.008)	0.62 [0.49, 0.73]	[-0.012, 0.016]	0.005 (0.5)	0.014 (1.4)	0.12
Sw%	0.971 (0.014)	0.974 (0.014)	0.62 [0.48, 0.73]	[-0.021, 0.026]	0.009 (0.9)	0.024 (2.5)	0.14
DS%	0.931 (0.030)	0.930 (0.029)	0.64 [0.50, 0.74]	[-0.050, 0.048]	0.018 (1.9)	0.049 (5.2)	0.79
St%	0.972 (0.012)	0.973 (0.014)	0.69 [0.57, 0.78]	[-0.019, 0.021]	0.007 (0.7)	0.020 (2.0)	0.39
**Intra-session reliability results of SI2 at fast walking**							
Sa	0.982 (0.008)	0.982 (0.008)	0.56 [0.41, 0.68]	[-0.014, 0.015]	0.005 (0.5)	0.015 (1.5)	0.55
Sw	0.973 (0.011)	0.973 (0.013)	0.51 [0.35, 0.64]	[-0.023, 0.023]	0.008 (0.8)	0.023 (2.3)	0.71
DS	0.902 (0.045)	0.907 (0.040)	0.56 [0.41, 0.68]	[-0.072, 0.082]	0.028 (3.1)	0.077 (8.5)	0.75
St	0.974 (0.012)	0.974 (0.011)	0.40 [0.22, 0.55]	[-0.025, 0.024]	0.009 (0.9)	0.025 (2.5)	0.97
SL	0.971 (0.015)	0.971 (0.015)	0.74 [0.64, 0.82]	[-0.020, 0.022]	0.008 (0.8)	0.021 (2.1)	0.75
GS	0.969 (0.015)	0.970 (0.015)	0.73 [0.62, 0.81]	[-0.021, 0.023]	0.008 (0.8)	0.022 (2.2)	0.48
Sa%	0.983 (0.007)	0.984 (0.008)	0.61 [0.47, 0.72]	[-0.013, 0.014]	0.005 (0.5)	0.013 (1.3)	0.41
Sw%	0.975 (0.010)	0.975 (0.012)	0.60 [0.45, 0.71]	[-0.020, 0.020]	0.007 (0.7)	0.020 (2.0)	0.55
DS%	0.901 (0.045)	0.908 (0.040)	0.56 [0.42, 0.68]	[-0.070, 0.084]	0.028 (3.1)	0.078 (8.6)	0.51
St%	0.975 (0.011)	0.976 (0.011)	0.42 [0.25, 0.57]	[-0.023, 0.024]	0.008 (0.9)	0.023 (2.4)	0.46

TABLE S4. Intra-session reliability results for symmetry index SI3 in 101 healthy adults (55 men) at preferred, dual-task, and fast walking speeds. The index values were obtained by applying Formula 3 to *Stance time* (Sa), *Swing time* (Sw), *Double-Support time* (DS), *Step time* (St), their respective percentages (Sa%, Sw%, DS%, and St%) of Sr, *Stride length* (SL), and to *Gait speed* (GS).

	Trial1: mean (SD)	Trial2: mean (SD)	ICC(2.1) [95% CI]	LOA [95% CI]	SEM (SEM%)	MDC (MDC%)	*p*
**Intra-session reliability results of SI3 at preferred walking**							
Sa	-0.01 (0.38)	-0.05 (0.38)	0.83 [0.76, 0.88]	[-0.48, 0.38]	0.16 (-516.5)	0.43 (-1431.7)	0.37
Sw	-0.01 (0.63)	0.07 (0.61)	0.81 [0.73, 0.87]	[-0.65, 0.81]	0.27 (976.6)	0.74 (2707.0)	0.37
DS	0.66 (2.04)	0.60 (1.91)	0.91 [0.87, 0.94]	[-1.71, 1.61]	0.60 (94.5)	1.65 (262.0)	0.85
St	0.13 (0.68)	0.22 (0.63)	0.80 [0.72, 0.86]	[-0.72, 0.89]	0.29 (165.5)	0.81 (458.8)	0.35
SL	-0.21 (0.97)	-0.27 (0.99)	0.93 [0.90, 0.95]	[-0.78, 0.66]	0.26 (-109.9)	0.73 (-304.7)	0.64
GS	-0.22 (0.98)	-0.28 (0.99)	0.94 [0.91, 0.96]	[-0.74, 0.61]	0.25 (-97.5)	0.68 (-270.2)	0.59
Sa%	0.00 (0.38)	-0.05 (0.37)	0.83 [0.75, 0.88]	[-0.48, 0.36]	0.16 (-617.0)	0.43 (-1710.2)	0.28
Sw%	-0.01 (0.64)	0.09 (0.62)	0.84 [0.76, 0.89]	[-0.60, 0.79]	0.26 (612.7)	0.71 (1698.2)	0.29
DS%	0.64 (2.06)	0.59 (1.94)	0.90 [0.86, 0.93]	[-1.78, 1.69]	0.62 (101.2)	1.73 (280.7)	0.88
St%	0.13 (0.67)	0.22 (0.63)	0.81 [0.73, 0.87]	[-0.68, 0.86]	0.28 (161.0)	0.78 (446.3)	0.32
**Intra-session reliability results of SI3 at dual-task walking**							
Sa	0.01 (0.48)	-0.06 (0.46)	0.69 [0.57, 0.78]	[-0.79, 0.65]	0.26 (-980.3)	0.72 (-2717.3)	0.30
Sw	-0.03 (0.77)	0.09 (0.76)	0.78 [0.69, 0.85]	[-0.85, 1.09]	0.36 (1145.1)	0.99 (3174.1)	0.27
DS	0.39 (1.91)	0.70 (2.09)	0.85 [0.77, 0.90]	[-1.79, 2.43]	0.79 (144.2)	2.18 (399.8)	0.26
St	0.10 (0.81)	0.28 (0.80)	0.72 [0.59, 0.80]	[-0.98, 1.34]	0.43 (228.8)	1.20 (634.3)	0.12
SL	-0.26 (1.21)	-0.24 (1.21)	0.95 [0.92, 0.96]	[-0.76, 0.80]	0.28 (-112.6)	0.77 (-312.2)	0.88
GS	-0.23 (1.21)	-0.26 (1.23)	0.94 [0.91, 0.96]	[-0.89, 0.84]	0.31 (-124.8)	0.86 (-345.8)	0.89
Sa%	0.01 (0.46)	-0.05 (0.46)	0.75 [0.66, 0.83]	[-0.68, 0.57]	0.23 (-1020.1)	0.63 (-2827.5)	0.38
Sw%	-0.01 (0.78)	0.09 (0.79)	0.76 [0.67, 0.83]	[-0.96, 1.15]	0.38 (1041.0)	1.06 (2885.6)	0.38
DS%	0.37 (1.94)	0.71 (2.07)	0.85 [0.77, 0.90]	[-1.74, 2.42]	0.78 (145.1)	2.17 (402.2)	0.23
St%	0.10 (0.78)	0.25 (0.78)	0.79 [0.69, 0.86]	[-0.81, 1.11]	0.36 (199.5)	0.99 (553.1)	0.18
**Intra-session reliability results of SI3 at fast walking**							
Sa	-0.05 (0.49)	-0.10 (0.47)	0.79 [0.71, 0.85]	[-0.65, 0.55]	0.22 (-298.8)	0.61 (-828.2)	0.44
Sw	0.03 (0.70)	0.13 (0.72)	0.82 [0.75, 0.88]	[-0.71, 0.91]	0.30 (394.6)	0.83 (1093.8)	0.31
DS	1.04 (2.64)	1.04 (2.51)	0.75 [0.65, 0.82]	[-3.59, 3.60]	1.29 (124.0)	3.57 (343.6)	0.98
St	0.20 (0.67)	0.34 (0.65)	0.60 [0.46, 0.72]	[-1.00, 1.28]	0.42 (153.9)	1.16 (426.5)	0.13
SL	-0.15 (0.92)	-0.16 (0.92)	0.90 [0.86, 0.93]	[-0.81, 0.79]	0.29 (-189.9)	0.80 (-526.4)	0.94
GS	-0.16 (0.93)	-0.17 (0.93)	0.89 [0.84, 0.93]	[-0.86, 0.84]	0.30 (-180.0)	0.85 (-499.0)	0.94
Sa%	-0.03 (0.47)	-0.09 (0.46)	0.81 [0.73, 0.87]	[-0.61, 0.49]	0.20 (-326.0)	0.56 (-903.6)	0.37
Sw%	0.05 (0.70)	0.13 (0.71)	0.82 [0.74, 0.87]	[-0.74, 0.91]	0.30 (342.4)	0.83 (949.1)	0.41
DS%	1.01 (2.71)	1.03 (2.52)	0.74 [0.64, 0.82]	[-3.70, 3.75]	1.34 (130.8)	3.70 (362.5)	0.95
St%	0.22 (0.67)	0.32 (0.63)	0.65 [0.52, 0.75]	[-0.96, 1.16]	0.39 (142.0)	1.07 (393.7)	0.29

TABLE S5. Intra-session reliability results of dual-task cost indices, DTC and DTC%, in 101 healthy adults (55 men). These indices are obtained for *Stride time* (Sr), *Stance time* (Sa), *Swing time* (Sw), *Double-Support time* (DS), *Step time* (St), their respective percentages (Sa%, Sw%, DS%, and St%) of Sr, *Stride length* (SL), *Gait speed* (GS), *Cadence* (Cad), SL and GS normalized to leg length (SL_{n1} and GS_{n1}) and to body height (SL_{n2} and GS_{n2}).

	Trial1: mean (SD)	Trial2: mean (SD)	ICC(2.1) [95% CI]	LOA [95% CI]	SEM (SEM%)	MDC (MDC%)	*p*
**Intra-session reliability results of dual-task index DTC**							
Sr	0.051 (0.123)	0.045 (0.103)	0.94 [0.92, 0.96]	[-0.080, 0.070]	0.03 (56.3)	0.08 (155.9)	0.96
Sa	0.036 (0.082)	0.032 (0.069)	0.94 [0.91, 0.96]	[-0.055, 0.048]	0.02 (55.0)	0.05 (152.5)	0.98
Sw	0.015 (0.041)	0.013 (0.033)	0.94 [0.91, 0.96]	[-0.027, 0.022]	0.01 (63.8)	0.02 (176.8)	0.99
DS	0.010 (0.023)	0.009 (0.020)	0.92 [0.89, 0.95]	[-0.017, 0.016]	0.01 (63.1)	0.02 (174.9)	0.95
St	0.025 (0.061)	0.023 (0.051)	0.95 [0.93, 0.97]	[-0.037, 0.032]	0.01 (52.2)	0.03 (144.8)	0.97
SL	-0.039 (0.098)	-0.042 (0.099)	0.92 [0.88, 0.95]	[-0.081, 0.073]	0.03 (-68.1)	0.08 (-188.7)	0.79
GS	-0.088 (0.202)	-0.087 (0.190)	0.96 [0.94, 0.97]	[-0.109, 0.112]	0.04 (-45.1)	0.11 (-125.1)	0.95
Cad	-0.036 (0.087)	-0.033 (0.076)	0.96 [0.94, 0.97]	[-0.044, 0.050]	0.02 (-48.7)	0.05 (-135.1)	0.99
Sa%	0.306 (1.036)	0.331 (0.991)	0.89 [0.84, 0.92]	[-0.907, 0.957]	0.33 (105.0)	0.93 (291.2)	0.62
Sw%	-0.306 (1.036)	-0.331 (0.991)	0.89 [0.84, 0.92]	[-0.957, 0.907]	0.33 (-105.0)	0.93 (-291.2)	0.62
DS%	0.296 (1.024)	0.313 (1.007)	0.90 [0.85, 0.93]	[-0.898, 0.931]	0.33 (107.7)	0.91 (298.6)	0.67
SL_{n1}	-0.045 (0.109)	-0.049 (0.110)	0.92 [0.88, 0.94]	[-0.091, 0.083]	0.03 (-66.6)	0.09 (-184.6)	0.81
GS_{n1}	-0.101 (0.225)	-0.100 (0.211)	0.96 [0.94, 0.97]	[-0.125, 0.128]	0.05 (-45.3)	0.13 (-125.5)	0.94
SL_{n2}	-0.023 (0.057)	-0.025 (0.058)	0.92 [0.88, 0.94]	[-0.048, 0.043]	0.02 (-67.3)	0.05 (-186.5)	0.78
GS_{n2}	-0.052 (0.119)	-0.052 (0.111)	0.96 [0.94, 0.97]	[-0.065, 0.067]	0.02 (-45.4)	0.07 (-125.7)	0.94
**Intra-session reliability results of dual-task index DTC%**							
Sr	4.911 (11.942)	4.372 (9.983)	0.94 [0.92, 0.96]	[-7.658, 6.579]	2.58 (55.5)	7.14 (153.9)	0.98
Sa	5.545 (12.811)	4.957 (10.736)	0.94 [0.92, 0.96]	[-8.360, 7.184]	2.81 (53.6)	7.80 (148.5)	1.00
Sw	3.926 (10.647)	3.271 (8.473)	0.94 [0.91, 0.96]	[-7.015, 5.706]	2.32 (64.6)	6.44 (179.0)	0.98
DS	8.086 (17.805)	7.422 (15.981)	0.93 [0.89, 0.95]	[-13.278, 11.952]	4.54 (58.6)	12.59 (162.4)	1.00
St	4.910 (11.780)	4.396 (9.963)	0.95 [0.93, 0.97]	[-7.127, 6.100]	2.40 (51.5)	6.64 (142.7)	0.97
SL	-2.485 (6.278)	-2.738 (6.347)	0.92 [0.88, 0.94]	[-5.274, 4.769]	1.81 (-69.2)	5.01 (-191.8)	0.79
GS	-5.811 (13.465)	-5.798 (12.534)	0.95 [0.93, 0.97]	[-7.734, 7.760]	2.77 (-47.8)	7.69 (-132.5)	0.89
Cad	-3.741 (8.918)	-3.435 (7.895)	0.96 [0.94, 0.97]	[-4.583, 5.196]	1.76 (-49.2)	4.89 (-136.3)	1.00
Sa%	0.500 (1.655)	0.535 (1.583)	0.89 [0.84, 0.92]	[-1.459, 1.530]	0.54 (103.6)	1.49 (287.1)	0.65
Sw%	-0.788 (2.778)	-0.869 (2.661)	0.89 [0.84, 0.93]	[-2.568, 2.406]	0.89 (-107.7)	2.47 (-298.6)	0.58
DS%	2.674 (8.298)	2.699 (8.116)	0.89 [0.84, 0.92]	[-7.552, 7.603]	2.71 (101.0)	7.53 (280.1)	0.78

TABLE S6. Reference values for spatiotemporal gait parameters and symmetry ratio SI4, and the results of the following parameter comparisons: (1) preferred walking (PW) vs dual-task walking (DTW), (2) PW vs fast walking (FW), and (3) DTW vs FW. These parameters are *Stride time* (Sr) [s], *Stance time* (Sa) [s], *Swing time* (Sw) [s], *Double-Support time* (DS) [s], *Step time* (St) [s], their respective percentages (Sa%, Sw%, DS%, and St%) of Sr, *Stride length* (SL) [m], *Gait speed* (GS) [m/s], their respective normalized values to leg length (SL_{n1} [dimensionless] and GS_{n1} [s^{-1}]) and to body height (SL_{n2} [dimensionless] and GS_{n2} [s^{-1}]), and *Cadence* (Cad) [strides/s].

	PW: trials 1&2 mean (SD)	DTW: trials 1&2 mean (SD)	FW: trials 1&2 mean (SD)	PW vs DTW *p*	PW vs FW *p*	DTW vs FW *p*
**Reference values for spatiotemporal gait parameters and their comparisons**						
Sr	1.041 (0.054)	1.090 (0.124)	0.886 (0.064)	**0.003**	**<0.00001**	**<0.00001**
Sa	0.652 (0.042)	0.685 (0.083)	0.532 (0.043)	**0.002**	**<0.00001**	**<0.00001**
Sw	0.390 (0.021)	0.404 (0.044)	0.354 (0.026)	**0.042**	**<0.00001**	**<0.00001**
DS	0.131 (0.019)	0.141 (0.026)	0.089 (0.015)	**0.004**	**<0.00001**	**<0.00001**
St	0.521 (0.027)	0.545 (0.061)	0.443 (0.032)	**0.002**	**<0.00001**	**<0.00001**
SL	1.527 (0.139)	1.487 (0.158)	1.777 (0.169)	0.053	**<0.00001**	**<0.00001**
GS	1.471 (0.148)	1.383 (0.223)	2.016 (0.242)	**0.001**	**<0.00001**	**<0.00001**
Cad	0.963 (0.048)	0.929 (0.092)	1.135 (0.086)	**0.001**	**<0.00001**	**<0.00001**
Sa%	62.5 (1.5)	62.9 (1.6)	60.0 (1.4)	0.150	**<0.00001**	**<0.00001**
Sw%	37.5 (1.5)	37.1 (1.6)	40.0 (1.4)	0.150	**<0.00001**	**<0.00001**
DS%	12.5 (1.5)	12.8 (1.6)	9.9 (1.4)	0.170	**<0.00001**	**<0.00001**
SL_{n1}	1.721 (0.140)	1.674 (0.151)	2.002 (0.174)	**0.023**	**<0.00001**	**<0.00001**
GS_{n1}	1.659 (0.166)	1.559 (0.240)	2.275 (0.283)	**0.001**	**<0.00001**	**<0.00001**
SL_{n2}	0.895 (0.067)	0.871 (0.076)	1.041 (0.075)	**0.017**	**<0.00001**	**<0.00001**
GS_{n2}	0.863 (0.084)	0.811 (0.125)	1.182 (0.126)	**0.001**	**<0.00001**	**<0.00001**
**Reference values for symmetry ratio SI4 and their comparisons**						
Sa	1.001 (0.011)	1.001 (0.014)	1.003 (0.014)	0.979	0.410	0.429
Sw	1.000 (0.019)	1.000 (0.023)	0.998 (0.021)	0.951	0.650	0.638
DS	0.984 (0.060)	0.987 (0.060)	0.975 (0.076)	0.718	0.363	0.219
St	0.995 (0.020)	0.995 (0.024)	0.992 (0.018)	0.756	0.457	0.607
SL	1.008 (0.030)	1.009 (0.038)	1.005 (0.028)	0.973	0.505	0.476
GS	1.009 (0.031)	1.009 (0.038)	1.006 (0.029)	0.948	0.521	0.545
Sa%	1.001 (0.011)	1.001 (0.013)	1.002 (0.014)	0.996	0.487	0.517
Sw%	0.999 (0.019)	0.999 (0.023)	0.998 (0.021)	0.919	0.645	0.607
DS%	0.984 (0.060)	0.987 (0.060)	0.975 (0.076)	0.779	0.342	0.231
St%	0.995 (0.020)	0.995 (0.023)	0.992 (0.019)	0.933	0.270	0.312
